# Mechanically Interlocked Indigo Photoswitches

**DOI:** 10.1002/anie.4283917

**Published:** 2026-06-30

**Authors:** Alexander M. Wilmshurst, Taegeun Jo, Rebecca L. Kerridge, Akanksha Ashok Sangolkar, Zhihao Ling, Alex Buchanan, Neil Wells, Yael Ben‐Tal, Stefano Crespi, George T. Williams

**Affiliations:** ^1^ School of Chemistry and Chemical Engineering University of Southampton Southampton UK; ^2^ Department of Chemistry – Ångström Laboratory Uppsala University Uppsala Sweden; ^3^ Department of Chemistry University of British Columbia Vancouver Canada; ^4^ Institute For Life Sciences University of Southampton Southampton UK

**Keywords:** photoswitch, rotaxane, supramolecular chemistry

## Abstract

Photoswitches provide the opportunity to remotely and precisely control matter on the nanoscopic scale. For many materials and biological applications, photoswitches with long wavelength response are essential; however, few switches offer inherent response to red/near infra‐red light. Previous works have described the use of intermolecular interactions as a method to redshift the activation wavelength of photoswitches and to improve thermal half‐life. However, these systems are limited in their application due to the inherent bimolecularity of this strategy preventing its use in dilute or complex environments. Herein, we describe the use of topologically constrained supramolecular interactions to improve the switching properties of an indigo photoswitch within a [2]‐rotaxane. This enabled photoswitching with 730 nm light, as well as a 100‐fold increase in thermal half‐life and double the population of the metastable state under constant irradiation. This surpasses previous attempts at using supramolecular interactions to increase the thermal half‐life by >10‐fold. This novel strategy towards the redshifting and fine‐tuning of these molecular photoswitches has implications for the design of molecular machines and applied switching technologies. We anticipate that our insights into the design of such molecules will unlock new applications for mechanically interlocked molecules.

## Graphical abstract

By incorporating indigo photoswitches into the axle of a rotaxane, which features a hydrogen bonding macrocycle, we demonstrate improvement in activation wavelength, photostationary states and thermal reversion, an effect that is governed by topologically constrained supramolecular interactions.

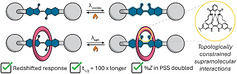

## 1 Introduction

Photoswitchable molecules enable the remote control of molecular structure and properties using light; these compounds underpin a range of technologies in diverse fields including biomedicine [1, 2, 3], energy storage [4, 5, 6, 7, 8, 9, 10], soft robotics [11, 12, 13], visual displays and optogenetics [14, 15, 16, 17]. The development of these applications has come hand in hand with the discovery of a range of switching architectures, with key classes including spiropyrans [18], donor‐acceptor Stenhouse adducts [19, 20], diarylethylenes [21], azobenzenes [22], heteroaryl [23, 24], and BF_2_‐azo compounds [25], hydrazones [3, 10, 17, 26, 27], aldimines [28, 29, 30], stiff‐stilbenes [31], phosphoalkenes [32] and the various subclasses of indigoid photoswitches, including hemiindigos [33], hemithioindigos [34], hemiphosphoindigos [35], iminothioindoxyls [36, 37], aurones [38] and (thio)indigo [39, 40, 41, 42, 43]. Considerable effort has been dedicated to designing such compounds that respond to long‐wavelength light; this has traditionally focused on synthetic modification of the photoswitch core, perhaps best exemplified by ortho‐substitution of azobenzenes [22, 44, 45, 46]. Regarding indigoid photoswitches, Dube and co‐workers have made great strides towards the near‐infrared regime by extending the conjugation of thioindigo photoswitches [47, 48, 49]. This strategy is of particular relevance for switches in biological and material applications [50, 51], since longer wavelengths offer deeper sample penetration and decreased phototoxicity [52].

Although indigo photoisomerisation was discovered in the 1950s [53], detailed and systematic investigation of indigo photoswitches occurred much later. *N,N′*‐alkylated indigo photoswitches were demonstrated by Hecht and co‐workers to offer red‐light response; however, the thermal half‐life of these species was incredibly short (< 5 s for *N*,*N*′‐dimethylindigo) [43]. Alternative substitution with aryl‐groups, achieved half‐lives of > 400 min whilst retaining response to 660 nm light. Subsequent synthetic advancements by Huang and co‐workers have expanded the repertoire of available indigo photoswitches, incorporating additional functional moieties, whilst retaining response to red light [41, 42]. These same authors in collaboration with the Priimägi group have also demonstrated that indigo photoswitches can be incorporated into polymer films, bridging the gap between solution and solid state photoswitching; however, their ability to switch efficiently is dependent on the nature of the *N*‐functionalisation and the polymer properties [54].

**FIGURE 1:**
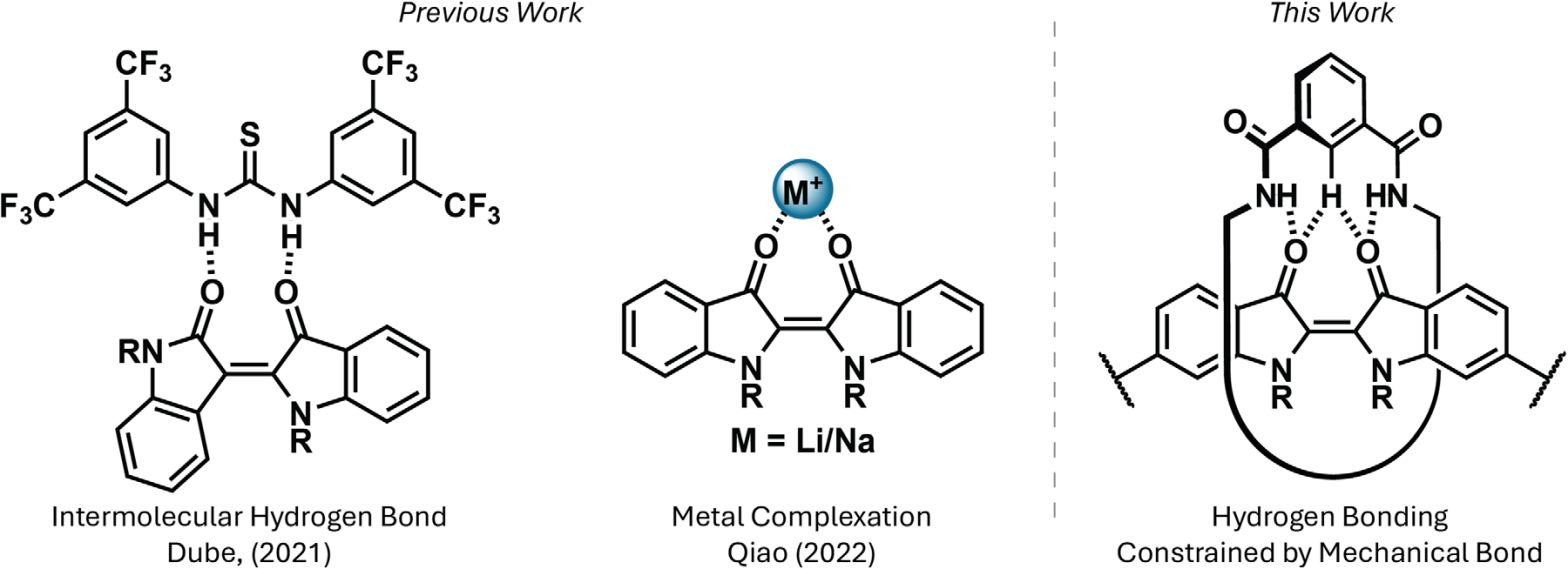
(Left) Previous work by Dube and co‐workers using bimolecular hydrogen bonding to improve photoswitch properties. (Middle) Previous work by Qiao and co‐workers investigating supramolecular interactions with metal ions to tune indigo relaxation dynamics and (Right) this work describing the use of mechanical bond mediated hydrogen bonding to improve indigo photoswitch properties.

Whilst derivatisation of the indigo core has been demonstrated to affect its spectral characteristics [55], we sought to identify alternative methods to engineer redshifted response. Thumser et al. demonstrated that supramolecular interactions with Schreiner's thiourea organocatalyst (*N*,*N*′‐bis[5‐bis(trifluoromethyl)phenyl]‐thiourea) [56] were able to improve the photoswitching properties of indirubin photoswitches, offering increased photostationary states and all red‐light response, although they did not observe changes in the *Z*→*E* thermal back isomerisation, and quantum yields were moderately reduced, Figure 1 [57]. These effects were attributed to hydrogen bonding interactions of the thiourea and the two indirubin carbonyls in the metastable *Z‐*isomer. Considering indigo photoswitches, both Qiao and co‐workers and Nielsen and co‐workers have investigated stabilisation of the *Z*‐ isomer, using cooperative carbonyl‐cation interactions and redox‐triggered *π*–*π* interactions, respectively, yielding an observed increase in half‐life of the metastable state in both cases [58, 59].

Inspired by these works, we designed a [2]‐rotaxane with an indigo photoswitch embedded in the axle and a macrocycle featuring a diamide hydrogen bonding motif, Figure 1 [60]. The topological constraint inherent to the rotaxane structure enforces a high effective molarity between hydrogen bond donors on the macrocycle and the indigo carbonyls [61]. The resultant rotaxanes display a bathochromically shifted absorbance compared to the non‐interlocked axles, enabling switching in response to 730 nm light in the case of the dimethyl substituted indigo. We also observed a 100‐fold increase in thermal relaxation half‐life and double the population of the *Z‐*isomer in the photostationary state compared to the non‐interlocked axles. However, a concomitant decrease in the quantum yield of photoisomerisation for both rotaxanes was observed, indicating the likely effect of the mechanical bond on the potential energy surface (PES) of the switch.

## 2 Results and Discussion

**SCHEME 1:**
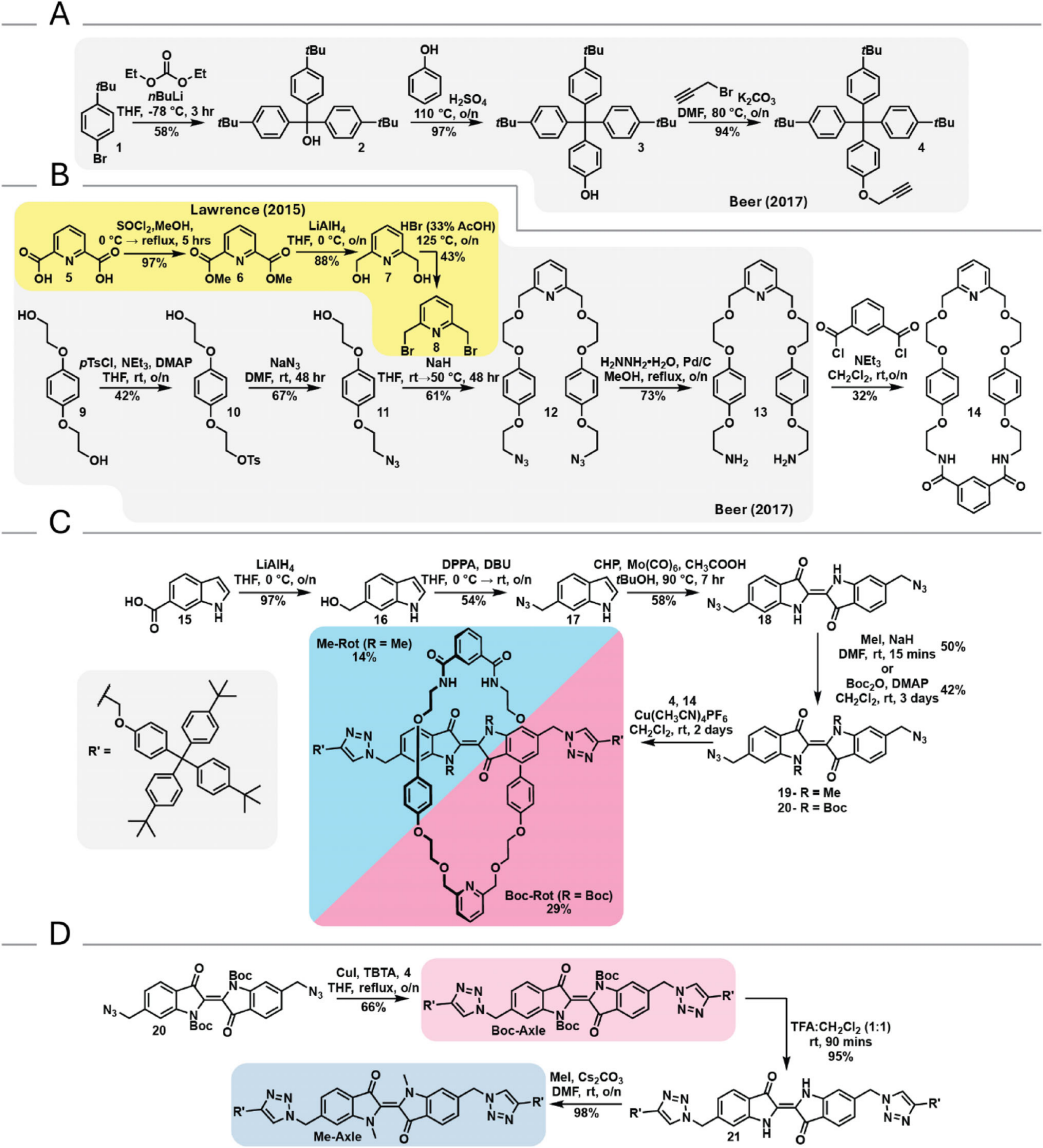
Synthetic route towards photoswitchable rotaxanes **Me‐Rot** and **Boc‐Rot**, and axles **Me‐Axle** and **Boc‐Axle**; full synthetic details are available in the Supporting Information.

Two rotaxanes were designed; each featured the same macrocycle, and thus, the same hydrogen bonding motif; however, the axles differed in the *N‐*substitution on the indigo. **Me‐Rot** features small *N,N′*‐dimethyl groups, designed to enable efficient supramolecular interactions between the indigo core of the axle and macrocycle, whilst **Boc‐Rot** was derivatised with bulky Boc‐groups to sterically minimise these interactions. The macrocycle featured a pyridine unit to enable the use of active template copper‐catalysed azide alkyne cycloaddition in the key mechanical bond‐forming step, with a diamide motif to act as a hydrogen bond donor [62, 63, 64]. To avoid the electronic influence of triazole, a methylene spacer was used to link tris‐(tert‐butylphenyl) stoppers [60]. The synthesis is outlined in Scheme 1, with full detail in the Supporting Information [60, 65].

**FIGURE 2:**
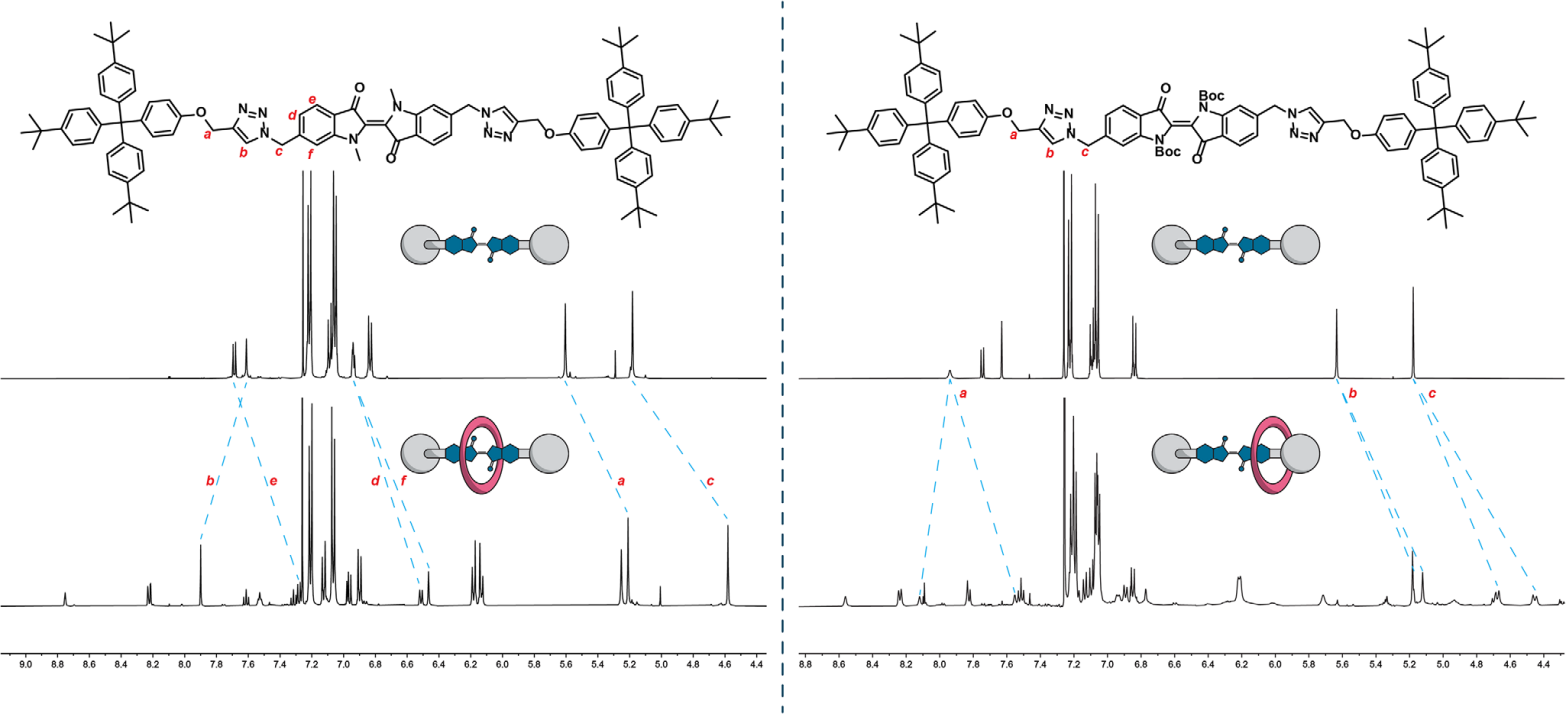
The ^1^H NMR spectra of methyl (left) and Boc (right) functionalised axles and rotaxanes.

NMR analysis of the two axle components and the rotaxanes confirms the mechanically interlocked structure (Figure 2). The splitting of signals a–c in the NMR of **Boc‐Rot** also presents evidence that the steric bulk of the two Boc groups prevents the macrocycle from crossing the central indigo core of the axle, desymmetrising the structure.

**FIGURE 3:**
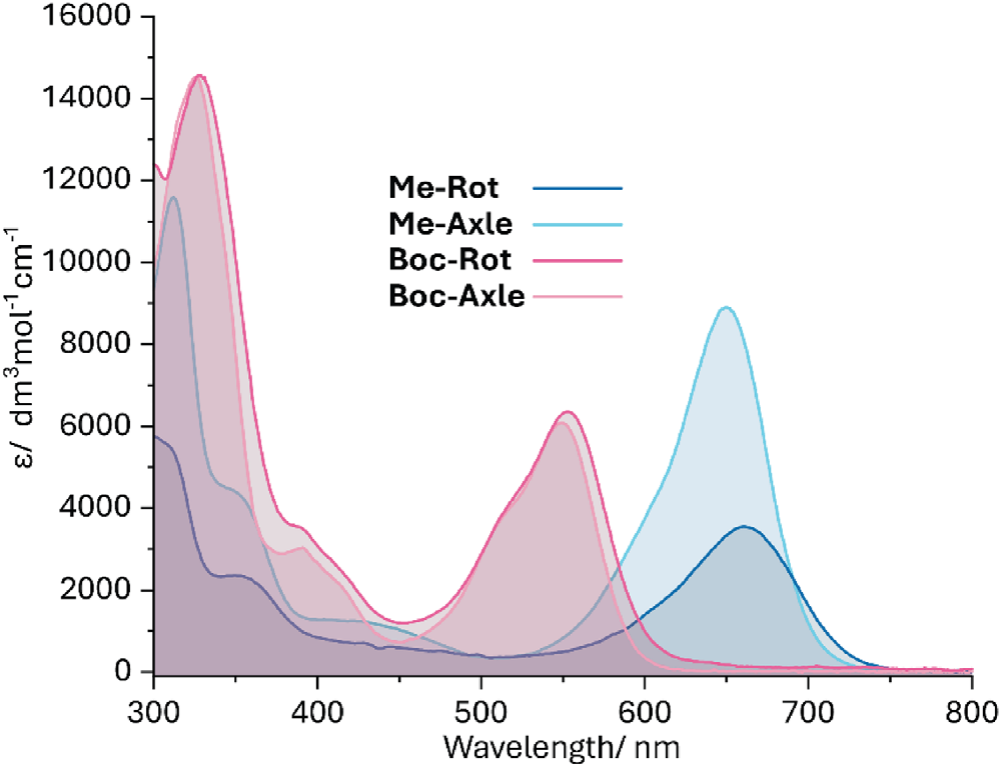
Molar absorption coefficients for dark equilibrated rotaxanes and axles in 50:50 MeCN:CHCl_3_.

The photophysical properties of the rotaxanes and axles were interrogated using UV‐vis spectroscopy in 50:50 MeCN:CHCl_3_ to ensure complete solubility, overviewed in Table 1. Unfortunately, at NMR relevant concentrations (1 mM) we did not observe any switching, precluding NMR being used for these experiments. As previously observed by Farka et al. [66], **Boc‐Axle** and **Boc‐Rot** featuring electron‐withdrawing Boc groups yielded blue‐shifted absorbance maxima compared to the methylated **Me‐Axle** and **Me‐Rot** (Figure 3). In both cases, the presence of the macrocycle causes a bathochromic shift of the absorbance maxima and a broadening of the peak; this is more pronounced in the case of **Me‐Rot** which displays a *λ*_max_ shift of 11 nm compared to the axle, whilst the *λ*_max_ of **Boc‐Rot** is only redshifted by 4 nm. We attribute this difference to the increased steric bulk of the *tert‐*butyl groups of **Boc‐Rot** preventing efficient hydrogen bonding of the macrocycle with the indigo carbonyls. Upon incorporation into the rotaxane structure, **Me‐Rot** also shows a drastic decrease in molar absorptivity coefficient at the *λ*_max_ by over 50% compared to **Me‐Axle**. This is both reduced and reversed in the case of the Boc‐functionalised indigos, with **Boc‐Rot** showing a slight increase (≈ 20%) in extinction coefficient.

**TABLE 1:** Key photoswitching parameters.

	*λ*_max_ *E* (nm)	*ε* at (*λ*_max_ *E*) (L mol^−1^ cm^−1^)	*λ*_max_ PSS (nm)	*ε* at (*λ*_max_ PSS) (L mol^−1^ cm^−1^)	%*Z* at the PSS at 25°C (*λ* LED)	*t*_1/2_ at 25°C (s)	*Φ*_(_ *_E→Z_*_)_ (LED)	*Φ*_(Z_ *_→E_*_)_ (LED)
**Me‐Rot**	661	4430	653	1830	10% 730 nm 19% 680 nm **40% 625 nm** 22% 590 nm 23% 565 nm 16% 530 nm 12% 505 nm 19% 470 nm	22.5	0.8% (625 nm)	38% (625 nm)
**Boc‐Rot**	553	6350	542	3740	0% 730 nm 0% 680 nm 21% 625 nm **44% 590 nm** 37% 565 nm 33% 530 nm 24% 505 nm 12% 470 nm	3570	2% (470 nm) 1% (590 nm)	4% (470 nm) 3% (590 nm)
**Me‐Axle**	650	8900	649	8970	0% 730 nm 0% 680 nm **20% 625 nm** 6% 590 nm 0% 565 nm 0% 530 nm 0% 505 nm 0% 470 nm	0.223	18% (625 nm)	3% (625 nm)
**Boc‐Axle**	549	6080	516	3500	0% 730 nm 0% 680 nm 46% 625 nm **55% 590 nm** 46% 565 nm 41% 530 nm 28% 505 nm 11% 470 nm	85.8	7% (470 nm) 5% (590 nm)	10% (470 nm) 1% (590 nm)

*Note*: Nominal wavelength of the LED that achieved the highest % of *Z*‐isomer in each case presented in bold.

The accumulation of the *Z*‐isomer of **Me‐Rot** at the PSS reached upon 730 nm irradiation was modest (10%). We sought to improve upon this by investigating other solvents; however, we were limited by the solubility of the compounds and were only able to perform measurements in dichloromethane and toluene. Unfortunately, there were no observed improvements, and, in fact, each of the alternative solvents resulted in a lower Z isomer %. The full results of these studies are tabulated in Table S1.

**FIGURE 4:**
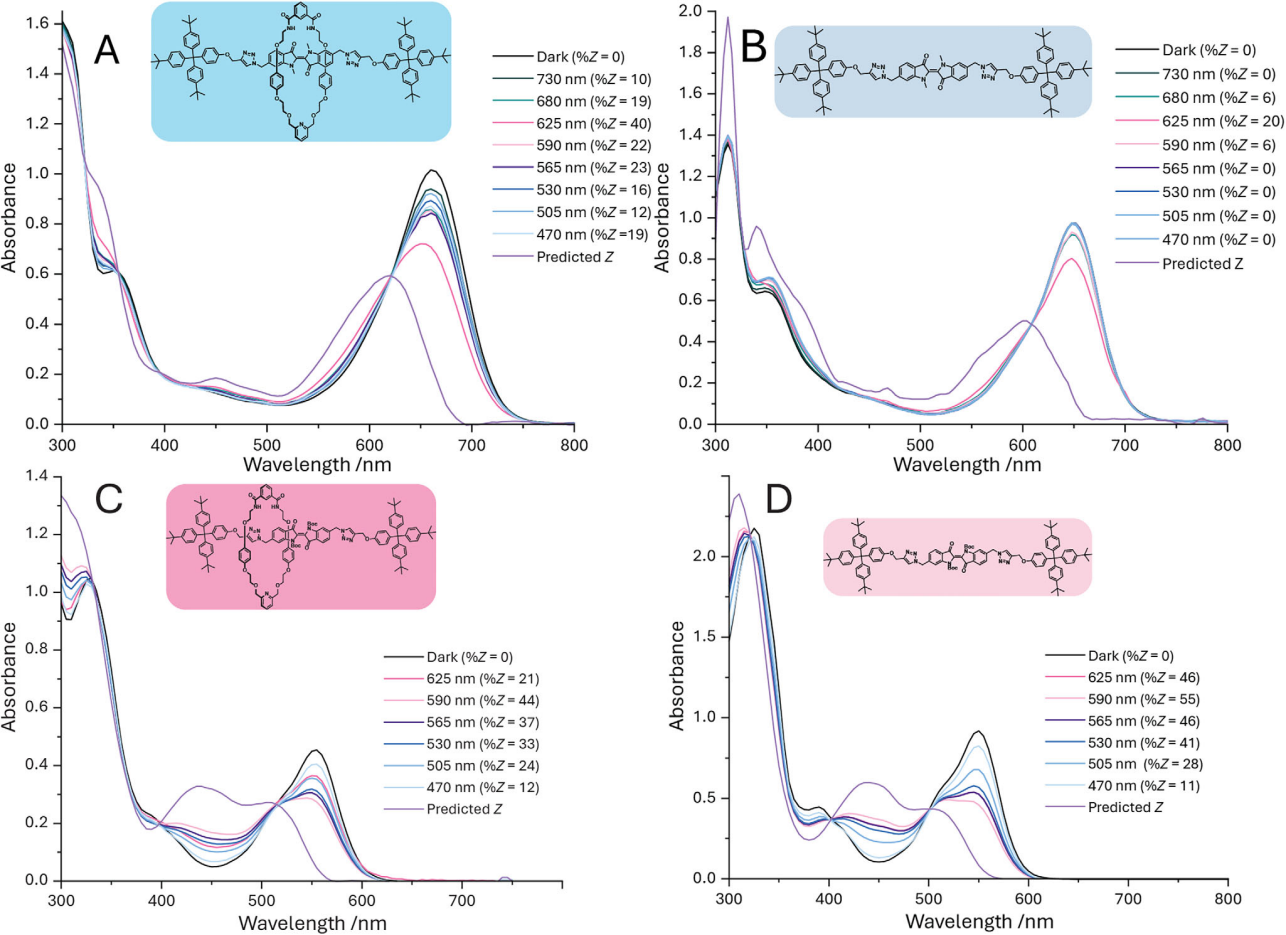
Absorbance spectra of the photostationary states of (a) **Me‐Rot** (220 µM), (b) **Me‐Axle** (100 µM), (c) **Boc‐Rot** (85 µM), and (d) **Boc‐Axle** (150 µM) in 50:50 MeCN:CHCl_3_ under constant LED irradiation. Data for LEDs that did not trigger isomerisation is omitted.

As anticipated, **Me‐Axle** and **Me‐Rot** displayed much shorter thermal half‐lives (0.223 and 22.5 s, respectively) than **Boc‐Axle** and **Boc‐Rot** (85.8 mins and 3570 s, respectively), in agreement with previous reports investigating *N‐*functionalisation effects on indigo photoswitches [43, 66]. Upon incorporation of the axles into the rotaxane structure, drastic decreases in rate of thermal reversion were observed, with the methylated system displaying a ≈100‐fold increase in thermal half‐life and the Boc‐functionalised, system displaying a ≈41‐fold increase (Figures S148, S151, S154, and S157). We attribute this in part to the topologically constrained rotaxane structure enforcing high relative molecularity of the hydrogen bond donors and the carbonyls of the indigo, since the macrocycle is unable to diffuse away. However, it should also be noted that the steric effects of the electron‐withdrawing Boc group would make the carbonyls on the indigo core poorer hydrogen bond acceptors, and both factors will play a role in changing the hydrogen bond strength and thus *t*_1/2_ [66]. This is supported by the energy‐minimised structures discussed later in the manuscript in which more stabilising hydrogen bonding interactions occur in **Me‐Rot** than **Boc‐Rot**. Such effects were not observed by Dube and co‐workers, and it cannot be ruled out that this may be an effect of the steric influence of the macrocycle yet were this to be the case, we would anticipate the Boc‐system to be more greatly affected. However, we were surprised to observe any impact at all, given the decreased proportion of the *Z‐*isomer in the photostationary states (Figure 4).

Following procedures outlined by Stranius and Börjesson [67], the quantum yields of the *E→Z* photoisomerisation of all four molecules were measured, as were quantum yields for **Boc‐Axle** and **Boc‐Rot** (Figures S161–166) for the *Z→E* photoisomerisation. These were studied using the wavelength of irradiation that tiggered the largest accumulation of the *Z‐*isomer in the PSS. The rapid thermal relaxation of **Me‐Axle** and **Me‐Rot** precluded studying Z*→E* photoisomerization for these compounds in the same way. In all cases, the incorporation of the **Boc‐Axle** into the rotaxane structure decreased the quantum yield, in line with a decrease in the %*Z* at the photostationary state observed in Figure 4. However, the story is not so simple in the case of **Me‐Axle** and **Me‐Rot**, since the Φ_(_ *_Z→E_*_)_ in **Me‐Rot** is substantially increased compared to **Me‐Axle,** whilst the opposite is true of the Φ_(_ *_E→Z_*_)_, yet an improved PSS is observed. This alludes to the multi‐faceted effect of the rotaxane structure on switching, which our groups continue to study.

An additional key parameter required for the translation of photoswitches into real‐world applications is their resistance to degradation upon repeated isomerisation cycles. Each compound was subject to 10 cycles of either photoisomerization then thermal relaxation, or photoisomerization in both directions. Degradation was found to be minimal in all cases (Figures S167–S170).

A control experiment was conducted using a “half‐macrocycle” formed consisting of a phenoxyethanamine‐derived isothalamide. Since this compound contains the same hydrogen bond donor unit as the macrocycles, we reasoned that it would act as a good model to establish the importance of the mechanical bond in tuning the photoswitching properties. With 10 equivalents of the half‐macrocycle added to solutions of the two axle components, there were no observable changes in the absorbance spectrum, thus wavelengths of activation were not shifted. We also observed a decreased % of Z‐isomer in the PSS compared to the relevant rotaxanes, reducing from 40% to 7% and 44% to 10% in the methyl and Boc functionalised systems, respectively. However, in both cases, we observed an increase in *t*_1/2_; in the case of the Boc‐Axle, the addition of the half‐macrocycle yielded a >2‐fold increase in stability compared to Boc‐Rot. We attribute this to the increased flexibility of the half‐macrocycle enabling more efficient hydrogen bonding with the Z‐isomer of the Boc‐substituted indigo, rather than the mechanically interlocked macrocycle. These results show that whilst the addition of hydrogen bond donor units is able to stabilise the Z‐isomer, the rotaxane structure is required to have an effect on the activation wavelength and to increase the accumulation of the metastable isomer.

**FIGURE 5:**
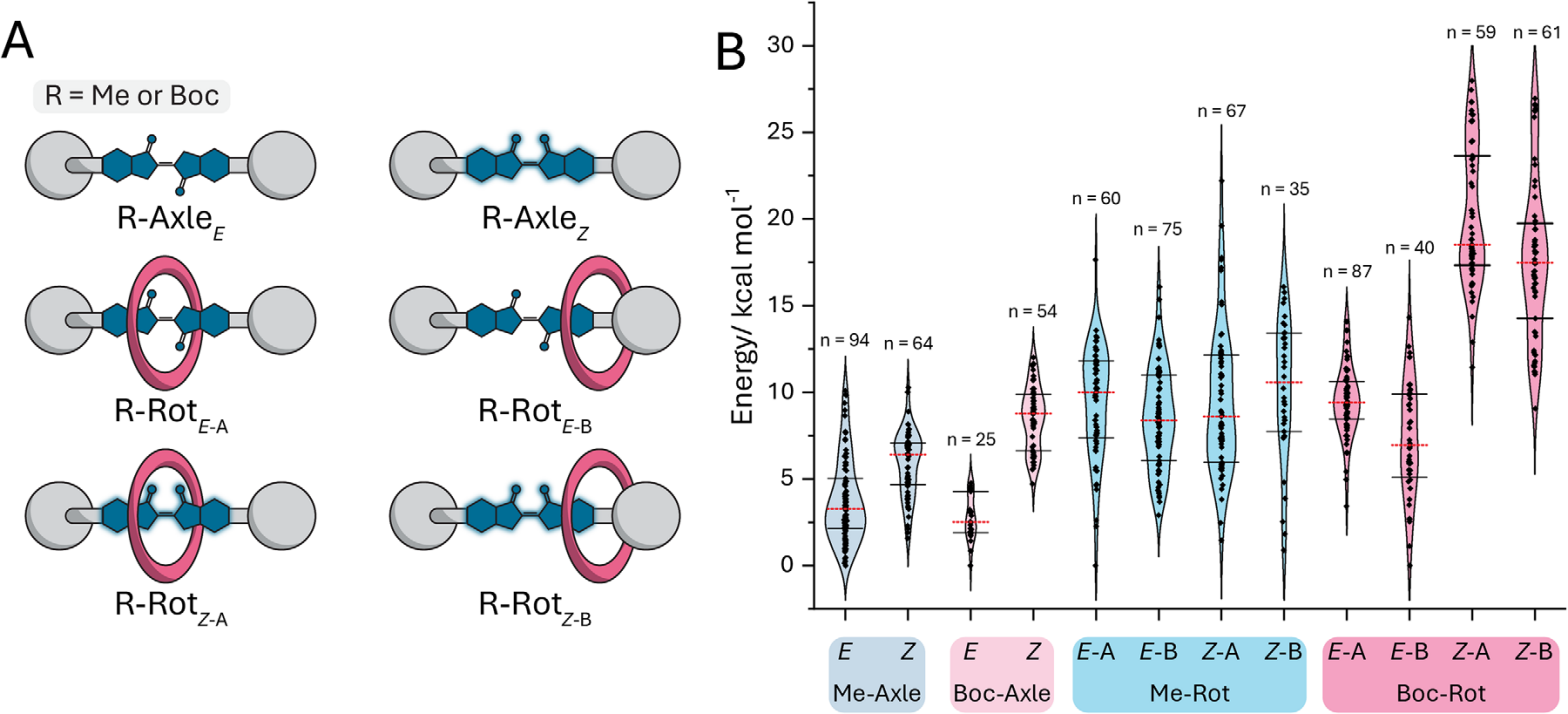
(A) The nomenclature denoting the configurational isomers of the compounds studied. (B) The violin plots showing the distribution of relative Gibbs free energies (Δ*G*, kcal/mol) for the conformers in the *E‐* and Z‐configurations of **Me‐Axle**, **Boc‐Axle**, **Me‐Rot** and **Boc‐Rot**. Each points represent the energies of distinct conformers, and *n* indicates the number of conformers obtained for each set. The energies are reported relative to the lowest energy conformer found for the entire family (both *E* and *Z*). The upper and lower solid lines indicate the interquartile range (Q1–Q3), and the dotted red lines indicate the median.

To better understand the supramolecular interactions between the macrocycle and the rotaxane thread, we performed computational conformational analysis following an established protocol (full details are provided in the Supporting Information: Section 5.1) [68, 69, 70, 71, 72, 73, 74]. The studied conformations are illustrated schematically in Figure 5A. The *E*‐ and *Z*‐isomers of each compound were subcategorised into *A* and *B* types, distinguished based on the interactions between the ring and the axle parts. Conformers containing hydrogen bonding between the amide protons of the macrocycle and the carbonyl oxygen atoms from the indigo in the axle with hydrogen‐oxygen distances shorter than 2.8 Å were classified as *A‐type*, whilst those that did not were classified *B*‐*type* (data given in Figures S177 and S178). The violin plots show the distribution of the Gibbs free energy for each conformer (Figure 5B). In the case of **Me‐Rot**, the Gibbs free energy distributions of all four sets (**Me‐Rot*_E_*_‐A_**, **Me‐Rot*_E_*_‐B_**, **Me‐Rot*_Z_*_‐A_**, and **Me‐Rot*_Z_*_‐B_**) range comparable energy windows, indicating no pronounced energetic preference among them. Consistently, the median Gibbs free energies for all sets fall within a narrow range of 8.4–10.6 kcal/mol. This behaviour contrasts with that observed for **Me‐Axle**, where the *Z*‐isomer exhibits a slightly higher overall energy distribution relative to the *E*‐isomer. In **Me‐Rot**, however, no clear energetic distinction between the *E‐* and *Z‐*isomer forms are shown, suggesting that the presence of the macrocycle significantly modifies the conformational energy landscape upon mechanical interlocking. Notably, the relative low energies window of the *Z*‐isomers for **Me‐Rot** may be correlated with the experimentally observed longer thermal half‐life of **Me‐Rot*_Z_*** compared with **Me‐Axle*_Z_***. Although no dominant energetic preference is observed between **Me‐Rot*_Z_*_‐A_** and **Me‐Rot*_Z_*_‐B_**, both conformer types remain energetically accessible within the close energy window. The distance of hydrogen‐bonding (N‐H···O) of conformers **Me‐Rot*_Z_*_‐A_** reveals that, in most conformers, two sets of hydrogen bonding were observed. This double hydrogen‐bonding pattern may suggest that the **Me‐Rot*_Z_*_‐A_** conformers contribute to inhibiting the isomerisation process by effectively locking the indigo core through the interactions.

In contrast, the introduction of the bulky Boc substituent leads to pronounced steric hindrance between the indigo core and the macrocycle, since *A‐type* conformers exhibit generally higher energy distributions than *B‐type* conformers, regardless of the *E*‐ or *Z*‐isomer configuration. This trend indicates that the Boc group weakens the formation of effective hydrogen bonds between the macrocycle and the indigo carbonyl groups. The reduced efficiency of the interaction is consistent with the lessened effect of the mechanical bond on the Boc‐substituted system compared to the Me‐substituted system, underscoring the importance of intramolecular interactions between macrocycle and axle. These computational results could highlight the influence of the mechanical bond on the PES. Steric hindrance in the Boc‐substituted system may not cause a substantial change in PES compared to the bare axle, resulting from nullifying the interaction between the mechanically interlocked components, while in the Me‐substituted rotaxane the macrocycle induces a significant change in energy distribution. To provide evidence for the importance of these supramolecular interactions, rather than a proton transfer process between the indigo carbonyl groups and the macrocycle, we turned to SF‐DFT calculations. In brief, it was determined that complete proton transfer was energetically unfavourable in both the ground (ca. 40 kcal mol^−1^ of activation energy) and excited states (17.7 kcal mol^−1^ of activation energy); however, the NH···O hydrogen bonds do play a key role in the switching of **Me‐Rot**; these results are discussed in detail in the Supporting Information (Section 5.2).

## 3 Conclusions

In summary, we have demonstrated that the mechanical bond can be used to create a unimolecular supramolecular system which improves the photoswitching properties of indigo photoswitches, and that the extent of the improvement can be tuned by *N‐*substitution. *N,N*’‐functionalising the indigo photoswitch with methyl groups allows efficient hydrogen bonding between macrocycle and indigo, whereas sterically demanding Boc groups inhibited these interactions. These interactions lead to a redshifted response to enable longer wavelength photoswitching, attained a higher % of *Z*‐isomer in the PSS on irradiation with all LEDs measured, and slowed *Z*→*E* thermal back isomerisation by two orders of magnitude, although there is a concomitant reduction in quantum yield in both directions. This represents the first strategy capable of not only redshifting response and increasing PSS but also extending the lifetime of the metastable state. This approach overcomes limitations of bimolecular systems, which use supramolecular interactions to tune photoswitching, opening avenues in functional systems that can be used in dilute concentrations in complex environments like cells. Work is currently underway within our laboratory to understand the effects of the dynamics of the system (i.e., macrocycle size and geometry), and to gain a better understanding of the effect of the macrocycle on the PES of these switches. This will allow us to more precisely tune properties, towards applications in a range of other switching architectures, and will enable us to achieve far greater effects, for example, improved PSS.

## Author Contributions

**Alexander M. Wilmshurst**: investigation, data curation, writing – review and editing, validation, writing – original draft, formal analysis. **Taegeun Jo**: investigation, writing – review and editing, writing – original draft, validation, data curation, formal analysis, visualization. **Rebecca L. Kerridge**: investigation, writing – review and editing. **Akanksha Ashok Sangolkar**: investigation. **Zhihao Ling**: investigation, writing – review and editing. **Alex Buchanan**: investigation, writing – review and editing. **Neil Wells**: investigation, formal analysis, writing – review and editing. **Yael Ben‐Tal**: methodology, validation, writing – review and editing. **Stefano Crespi**: funding acquisition, writing – original draft, writing – review and editing, methodology, supervision, resources, formal analysis. **George T. Williams**: conceptualization, investigation, funding acquisition, writing – original draft, validation, writing – review and editing, project administration, supervision, resources.

## Conflicts of Interest

The authors declare no conflicts of interest.

## Supporting information



The authors have cited additional references within the Supporting Information [75, 76, 78, 79, 80, 81, 82, 83, 84, 85, 86, 87]. **Supporting File 1**: anie73217‐sup‐0001‐SuppMat.docx.

## Data Availability

The data that support the findings of this study are available in the Supporting Information of this article.
