# Supplementary material for: Mechanically Interlocked Indigo Photoswitches
Source: Angew Chem Int Ed Engl. 2026 Jun 30;65(34):e4283917. doi: 10.1002/anie.4283917 (PMC13480533; doi:10.1002/anie.4283917)
Supplement: Supplementary file 1 [file ANIE-65-e4283917-s001.docx]

Tion

Electronic Supporting Information

Mechanically Interlocked Indigo Photoswitches

Alexander M. Wilmshurst,^[a]^ Taegeun Jo,^[b]^ Rebecca L. Kerridge, ^[a]^ Akanksha Ashok Sangolkar,^[b]^ Zhihao Ling, ^[a]^ Alex Buchanan, ^[a]^ Neil Wells, ^[a]^ Yael Ben-Tal,^[c]^ Stefano Crespi^[b]^* & George T. Williams^[a,d]^*

[a] School of Chemistry and Chemical Engineering, University of Southampton, Southampton SO17 1BJ, UK.

[b] Department of Chemistry – Ångström Laboratory, Uppsala University, Regementsvägen 10, 752 37 Uppsala, Sweden.

[c] Department of Chemistry, University of British Columbia, Vancouver, British Columbia V6T 1Z1, Canada.

[d] Institute for Life Sciences (IfLS), University of Southampton, Southampton, SO17 1BJ, UK.

Table of Contents

[Table of Contents	1](#_Toc219055755)

[1	General Methods	4](#_Toc219055756)

[1.1	UV-Vis Spectrophotometer Experimental Set-up	6](#_Toc219055757)

[2	Synthesis and Characterisation	7](#_Toc219055758)

[2.1	Synthesis of 6-hydroxymethylindole, 16	7](#_Toc219055759)

[2.2	Synthesis of 6-azidomethylindole, 17	14](#_Toc219055760)

[2.3	Synthesis of 6,6’-bisazidomethylindigo, 18	21](#_Toc219055761)

[2.1	Synthesis of 6,6’-bis(azidomethyl)-*N*,*N*’-bis(methyl)indigo, 19	23](#_Toc219055762)

[2.1	Synthesis of 6,6’-bis(azidomethyl)-*N*,*N*’-bis(*tert*-butylcarbonyl)indigo, 20	30](#_Toc219055763)

[2.2	Synthesis of Dimethyl pyridine-2,6-dicarboxylate, 6	37](#_Toc219055764)

[2.3	Synthesis of 2,6-Pyridinedimethanol, 7	40](#_Toc219055765)

[2.4	Synthesis of 2,6-Bis(bromomethyl)pyridine, 8	43](#_Toc219055766)

[2.5	Synthesis of 2-(4-(2-hydroxyethoxy)phenoxy)ethyl 4-tosylate, 10	48](#_Toc219055767)

[2.6	Synthesis of 2-(4-(2-azidoethoxy)phenoxy)ethanol, 11	51](#_Toc219055768)

[2.7	Synthesis of 2,6-bis((2-(4-(2-azidoethoxy)phenoxy)ethoxy)methyl)pyridine, 12	54](#_Toc219055769)

[2.8	Synthesis of 2,2'-((((((pyridine-2,6-diylbis(methylene))bis(oxy))bis(ethane-2,1-diyl))bis(oxy))bis(4,1-phenylene))bis(oxy))bis(ethan-1-amine), 13	57](#_Toc219055770)

[2.9	Synthesis of 3,6,8,18,20,23-hexaoxa-11,15-diaza-1(2,6)-pyridina-7,19(1,4),13(1,3)-tribenzenacyclotetracosaphane-12,14-dione, 14	60](#_Toc219055771)

[2.10	Synthesis of Bis(4-(tert-butyl)phenyl)(4-isopropylphenyl)methanol, 2	68](#_Toc219055772)

[2.11	Synthesis of 4-(tris(4-(tert-butyl)phenyl)methyl)phenol, 3	71](#_Toc219055773)

[2.12	Synthesis of 4,4',4''-((4-(prop-2-yn-1-yloxy)phenyl)methanetriyl)tris(tert-butylbenzene), 4	73](#_Toc219055774)

[2.1	Synthesis of *N,N’*-Methyl Indigo [2]rotaxane, Me-Rot	75](#_Toc219055775)

[2.1	Synthesis of *N,N’*-Boc Indigo [2]rotaxane, Boc-Rot	85](#_Toc219055776)

[2.2	Synthesis of di-tert-butyl-3,3'-dioxo-6,6'-bis((4-((4-(tris(4-(tert-butyl)phenyl)methyl)phenoxy)methyl)-1H-1,2,3-triazol-1-yl)methyl)-[2,2'-biindolinylidene]-1,1'-dicarboxylate, Boc-Axle	95](#_Toc219055777)

[2.3	Synthesis of 6,6'-bis((4-((4-(tris(4-(tert-butyl)phenyl)methyl)phenoxy)methyl)-1H-1,2,3-triazol-1-yl)methyl)-[2,2'-biindolinylidene]-3,3'-dione, 21	102](#_Toc219055778)

[2.4	Synthesis of 1,1'-dimethyl-6,6'-bis((4-((4-(tris(4-(tert-butyl)phenyl)methyl)phenoxy)methyl)-1H-1,2,3-triazol-1-yl)methyl)-[2,2'-biindolinylidene]-3,3'-dione, Me-Axle	111](#_Toc219055779)

[3	UV vis characterisation data	120](#_Toc219055780)

[3.1	Molar absorption coefficients	120](#_Toc219055781)

[3.1.1	Procedure	120](#_Toc219055782)

[3.1.2	Dark Extinction Molar Absorption Coefficient	121](#_Toc219055783)

[3.1.3	Forward PSS Molar Absorption Coefficient	123](#_Toc219055784)

[3.1.4	Back PSS Molar Absorption Coefficient	125](#_Toc219055785)

[3.2	Photostationary states	126](#_Toc219055786)

[3.2.1	Procedure	126](#_Toc219055787)

[3.2.2	Spectra	128](#_Toc219055788)

[3.3	Forward Switching (*E* → *Z*)	130](#_Toc219055789)

[3.3.1	Procedure	130](#_Toc219055790)

[3.3.2	Spectra	131](#_Toc219055791)

[3.4	Back Switching (*Z* → *E*)	133](#_Toc219055792)

[3.4.1	Procedure	133](#_Toc219055793)

[3.4.2	Spectra	134](#_Toc219055794)

[3.5	Thermal reversion	136](#_Toc219055795)

[3.5.1	Procedure	136](#_Toc219055796)

[3.5.2	Spectra	137](#_Toc219055797)

[3.6	Quantum yield determination	139](#_Toc219055798)

[3.6.1	Actinometry	139](#_Toc219055799)

[3.6.2	Determining Quantum yields	141](#_Toc219055800)

[Fatigue studies	144](#_Toc219055801)

[3.6.3	Procedure	144](#_Toc219055802)

[3.6.4	Data	145](#_Toc219055803)

[4	Computational studies	147](#_Toc219055804)

[5	References	150](#_Toc219055805)

# 1 General Methods

Unless otherwise stated, all reagents were purchased from commercial sources and used without further purification.

Brine refers to a saturated aqueous solution of NaCl. THF refers to tetrahydrofuran. EDTA-NH_3_ solution refers to an aqueous solution of NH_3_ (17% w/w) saturated with sodium-ethylenediaminetetraacetate.

Flash column chromatography was performed using Biotage Isolera-4 automated chromatography system, employing Biotage SNAP for reverse phase chromatography or ZIP cartridges for normal phase chromatography. Analytical TLC was performed on precoated silica gel plates (0.25 mm thick, 60F254, Merck, Germany) and observed under UV light (254 nm) or visualised with KMnO_4_ unless otherwise stated.

Low resolution mass spectrometry was carried out by the mass spectrometry services at University of Southampton (Waters TQD mass spectrometer equipped with a triple quadrupole analyser with UHPLC injection [BEH C18 column; MeCN -H_2_O gradient 0.2% formic acid)]). High resolution mass spectrometry was carried out by the mass spectrometry services at the University of Southampton (MaXis, Bruker Daltonics, with a Time of Flight (TOF) analyser; samples were introduced to the mass spectrometer via a Dionex Ultimate 3000 autosampler and uHPLC pump in a gradient of 20% MeCN in *n*-hexane to 100% MeCN(0.2% formic acid) over 5-10 min at 0.6 mL/min; column: Acquity UPLC BEH C18 (Waters) 1.7 micron 50 × 2.1mm) or by the mass spectrometry services at the University of Warwick (Bruker MaXis (ESI-HR-MS) mass spectrometer, electrospray ionisation using direct injection from MeOH solution of samples doped with small amounts of CH_2_Cl_2_).

NMR spectra were recorded on Bruker AVIIIHD500 instruments at a constant temperature of 298 K. Chemical shifts are reported in parts per million from low to high field, referenced to residual solvent and assigned based on COSY, NOESY, HSQC and HMBC. Coupling constants (*J*) are reported in Hertz (Hz). ^1^H NMR was acquired at 500 MHz, ^13^C NMR was acquired at 101 MHz, ^19^F NMR was acquired at 376 MHz. Standard abbreviations indicating multiplicity were used as follows: m = multiplet, quint = quintet, q = quartet, t = triplet, d = doublet, s = singlet, app. = apparent, br = broad.

All melting points were determined using Electrothermal Standard Digital Melting Point Apparatus 100 240 Vac 50 60 Hz.

Fourier Transform Infrared Spectroscopy (FTIR) was performed on a Nicolet iS5 FT-IR Spectrometer supplied by Thermo Scientific. All wavenumbers are reported in reciprocal centimetres (cm^-1^). Intensity is reported in percentage (%) transmittance.

All UV-Vis spectroscopy was performed on a Cary Series 60 UV-Vis Spectrometer maintained at 25 °C by a Cary Single Cell Peltier Accessory – both supplied by Agilent Technology. All wavelengths are reported in nanometres (nm). Absorbance is reported in absorbance units (a.u.). All samples were analysed in 3500 µL Macro Fluorescence Cuvette with Stopper, 10 mm Path Length supplied by Thorlabs.

All data was generated using the Agilent Cary WinUV application suite, processed in Microsoft Excel, before plotting and analysis using OriginPro 2020.

Samples were irradiated by Mounted LED series powered by the T-Cube™ LED Driver – both provided by Thorlabs. The spectral ranges and item number of each LED are shown below:

Figure S1 – Reported spectral output and nominal wavelength Mounted LEDs provided by Thorlabs with normalised intensities.^[1]^

## 1.1 UV-Vis Spectrophotometer Experimental Set-up

In-situ measurements were performed by constant LED irradiation perpendicular to the measurement beam within the Cary 60. This was made possible by drilling a 10 mm diameter hole through the non-water jacketed side of the peltier sample holder; this experimental set up can be seen in figure S2.

Figure S2 - The experimental set up of the temperature-controlled UV-Vis spectrophotometer used along with the in-situ irradiation perpendicular to the spectrophotometer beam.

# 2 Synthesis and Characterisation

## 2.1 Synthesis of 6-hydroxymethylindole, 16

Indole-6-carboxylic acid (**15**) (5.0 g, 31.0 mmol, 1.0 eq.) was dissolved in dry THF (100 mL) under an argon atmosphere and cooled to 0 °C. LiAlH_4_ sln. (2.4 M, 27.0 mL, 65.2 mmol, 2.1 eq.) was added dropwise to the solution. The reaction was left stirring overnight. The reaction mixture was diluted dropwise with EtOAc (40 mL) at 0 °C followed by addition of methanol (7 mL) and DI H_2_O (7 mL) and stirred vigorously for 30 mins. The mixture was filtered through celite. The filtrate was then dried with MgSO_4_ and the solvent was removed *in vacuo* at room temperature to avoid degradation. The crude material was taken forward without further purification, a brown oil – 6-hydroxymethylindole, **16** — which solidified when upon standing (4.41 g, 30.0 mmol, 97%)

For analytical purposes, a sample of the material was purified by flash column chromatography (EtOAc:Hexane, 0% → 20% → 40%). Yields a yellow oil which solidifies when left to stand. When exposed to heat >35 °C, the product partially degrades and the appearance changes from yellow to brick red.

**16** has previously been reported,^[2, 3]^ but not fully characterised.

**^1^H NMR** (500 MHz, CDCl3) δ 8.28 (br s, 1H, H***_i_***), 7.62 (d, J = 8.1 Hz, 1H, H***_d_***), 7.26 – 7.23 (m, 1H, H***_k_***), 7.16 (dd, J = 3.2, 2.4 Hz, 1H, H***_h_***), 7.09 (dd, J = 8.1, 1.4 Hz, 1H, H***_e_***), 6.54 (ddd, J = 3.1, 2.0, 1.0 Hz, 1H, H***_g_***), 4.74 (s, 2H, H***_b_***), 2.26 – 2.02 (s, 1H, H***_a_***).

**^13^C NMR** (126 MHz, CDCl_3_) δ 136.1 (C***_f_***), 135.0 (C_c_), 127.6 (C***_j_***), 124.8 (C_h_), 120.9 (C***_d_***), 119.6 (C***_e_***), 110.0 (C***_k_***), 102.6 (C_g_), 66.2 (C***_b_***).

**HR-ESI-MS**: *m/z* = 170.0571 [M+Na]^+^ calc. 170.0576 for C_9_H_9_NNaO

**FTIR (neat)**: ν_max_ 3399.55 (m), 3298.72 (br m), 2873.62 (w), 1451.44 (m), 1343.62 (m), 1063.56 (m)

**R.f.**: 0.38 (50% EtOAc:Hexane)

**M.p.**: 37.2 – 39.5 °C

Figure S3 - ^1^H NMR (CDCl_3_, 500 MHz) of **16**

Figure S4 - JMOD NMR (CDCl_3_, 126 MHz) of **16**

Figure S5 - HMBC NMR (CDCl_3_) of **16**

Figure S6 - HSQC NMR (CDCl_3_) of **16**

Figure S7 - COSY NMR (CDCl_3_) of **16**

Figure S8 – FTIR (neat) of **16**

## 2.2 Synthesis of 6-azidomethylindole, 17

**16** (0.315 g, 2.14 mmol, 1 eq.) was dissolved in dry THF ( mL) under an argon atmosphere and cooled to 0 °C. DPPA (0.555 mL,0.707 g,2.57 mmol, 1.2 eq.) and DBU (0.384 mL,0.391 g,2.57 mmol, 1.2 eq.) were then both added dropwise. The reaction mixture was stirred overnight and allowed to warm to rt.

The reaction mixture was then diluted with H_2_O (100 mL) and washed with CH_2_Cl_2_ (3 x 100 mL). The org layers were collected, washed with HCl sln. (1M, 250 mL), dried with MgSO_4_ and the solvent was removed *in vacuo*.

The crude material was purified by flash column chromatography (5% EtOAc:Hexane) to yield 6-azidiomethylindole, **17** (0.199g, 1.16 mmol, 54 %) a clear, yellow oil.

**^1^H NMR** (500 MHz, CDCl_3_) δ 8.19 (br s, 1H, H***_h_***), 7.66 (d, *J* = 8.1 Hz, 1H, H***_c_***), 7.36 (s, 1H, H***_j_***), 7.24 (t, *J* = 3.0 Hz, 1H, H***_g_***), 7.09 (dd, *J* = 8.1, 0.9 Hz, 1H, H***_d_***), 6.61 – 6.54 (m, 1H, H***_f_***), 4.44 (s, 2H, H***_a_***).

**^13^C NMR** (126 MHz, CDCl_3_) δ 135.8 (C***_e_***), 129.1 (C***_b_***), 127.9 (C***_i_***), 125.0 (C***_g_***), 121.2 (C***_c_***), 120.5 (C***_d_***), 111.0 (C***_j_***), 102.7 (C***_f_***), 55.6 (C***_a_***).

**HR-EI-MS**: *m/z* = 130.0650 [M – N_3_]^+•^ (100) calc. 130.0651 for C_9_H_8_N; *m/z* = 144.0680 [M – N_2_] ^+•^ (58) calc. 144.0682 for C_9_H_8_N_2_; *m/z* = 172.0743 [M] ^+•^ calc. 172.0743 for C_9_H_8_N_3_

m/z = 172.0743 (M^•+^, 40%), 144.0680 (58), 130.0650 (100)

**FTIR (neat)**: ν_max_ 3410.76 (br w), 2089.03 (s), 1452.12 (m), 1345.56 (m), 1246.10, (br m), 723.66 (s)

**R.f.**: 0.52 (20% EtOAc:Hexane)

Figure S9 - ^1^H NMR (CDCl_3_, 500 MHz) of **17**

Figure S10- JMOD NMR (CDCl_3_, 126 MHz) of **17**

Figure S11 - HMBC NMR (CDCl_3_) of **17**

Figure S12 - HSQC NMR (CDCl_3_) of **17**

Figure S13 - COSY NMR (CDCl_3_) of **17**

Figure S14 - FTIR (neat) of **17**

## 2.3 Synthesis of 6,6’-bisazidomethylindigo, 18

Procedure adapted from Yamamoto *et al*.^[4]^

**17** (399 mg, 2.32 mmol,1 eq.), CHP (80% w/w in cumine, 0.915 mL, 970 mg, 5.10 mmol, 2.2 eq.), Mo(CO)_6_ (6.12 mg, 0.00232 mmol, 1x10^-3^ eq.), and acetic acid (0.0133 mL,13.9 mg, 0.232 mmol, 0.1 eq.) were dissolved in *t*BuOH (5. 22 mL). The reaction mixture was heated to reflux (90 °C) and stirred overnight, during which the solution went from clear to yellow to blue to red. The reaction mixture was then filtered, and the residue washed with MeOH until the filtrate ran clear. The filter paper was left to dry and the residue – 6,6’-bisazidomethylindigo, **18** (251 mg, 0.674 mmol, 58%), a blue powder – collected. The material was taken forward without further purification.

Due to poor solubility, limited spectroscopic data was able to be acquired.

**LR-EI-MS**: *m/z* = 373.2 [M]^+•^ calc. 372.4 for C_18_H_12_N_8_O_2_

**FTIR (neat)**: ν_max_ 3353.62 (m), 2093.58 (br m), 1633.03 (m), 1607.74 (s), 1584.95 (w), 1444.84 (m), 1391.96 (w), 1322.72 (m), 1291.14 (m), 1242.61 (w), 1195.77 (m), 1170.67 (m), 1131.37 (m), 1110.38 (s), 1078.78 (m), 983.81 (w), 939.80 (m), 887.04 (w), 868.07 (m), 835.30 (w), 800.27 (m), 773.62 (s), 721.49 (m), 639.22 (w), 666.23 (m), 615.40 (w), 597.12 (w), 555.64 (m)

**M.p.**: > 400 °C

Figure S15 - FTIR (neat) of **18**

## 1.1 Synthesis of 6,6’-bis(azidomethyl)-*N*,*N*’-bis(methyl)indigo, 19

**18** (50 mg, 0.134 mmol, 1 eq.), sodium hydride (11.28 mg, 60% w/w, 0.282 mmol, 2.1 eq.) and iodomethane (33.51 μL, 76 mg, 0.537 mmol, 4 eq.) were dissolved in DMF (1 mL) at rt and stirred for 10 min. The reaction mixture was then diluted with EtOAc (100 mL), washed with water (100 mL) and brine (5 x 100 mL). The organic phase was collected and dried with MgSO_4_ and the solvent was removed *in vacuo* to give **19**, a blue solid (27 mg, 0.067 mmol, 50%).

**^1^H NMR** (500 MHz, CDCl_3_) δ 7.71 (d, *J* = 7.8 Hz, 2H, H***_d_***), 7.06 (dd, *J* = 1.3, 0.6 Hz, 2H, H***_i_***), 6.98 (ddt, *J* = 7.7, 1.3, 0.6 Hz, 2H, H***_c_***), 4.46 (s, 4H, H***_a_***), 3.60 (s, 6H, H***_j_***).

**^13^C NMR** (126 MHz, CDCl_3_) δ 184.6 (C***_f_***), 153.4 (C***_h_***), 143.1 (C***_e_***), 126.5 (C***_b_***), 124.4 (C***_d_***), 121.4 (C***_g_***), 120.9 (C***_c_***), 109.8 (C***_i_***), 54.9 (C***_a_***), 36.7 (C***_j_***).

**HR-ESI-MS**: *m/z* = 423.1285 [M+Na]^+^ calc. 423.1288 for C_20_H_16_N_8_NaO_2_

**FTIR (neat)**: ν_max_ 2919.40 (br w), 2849.83 (w), 2114.53 (s), 1639.52 (m), 1610.79 (s), 1528.35 (w), 1448.54 (m), 1428.68 (m), 1366.03 (m), 1331.87 (m), 1260.91 (br m), 1197.25 (m), 1086.15 (s), 1073.81 (s), 1007.87 (s), 928.03 (m), 854.47 (w) 807.69 (m), 764.08 (m), 744.56 (m), 687.88 (w), 638.25 (w), 575.45 (m)

**R.f.**: 0.39 (30% EtOAc in Hexanes). Blue spot visible to eye. Dark blue spot also visible on the baseline as majority of compound decomposes when subject to silica chromatography.

**M.p.**: Decomposed at 226.0 °C

Figure S16 - ^1^H NMR (CDCl_3_, 500 MHz) of **19**

Figure S17 - JMOD NMR (CDCl_3_, 126 MHz) of **19**

Figure S18 - HMBC NMR (CDCl_3_) of **19**

Figure S19 - HSQC NMR (CDCl_3_) of **19**

Figure S20 - COSY NMR (CDCl_3_) of **19**

Figure S21 - FTIR (neat) of **19**

## 1.1 Synthesis of 6,6’-bis(azidomethyl)-*N*,*N*’-bis(*tert*-butylcarbonyl)indigo, 20

**18** (370 mg, 0.994 mmol, 1 eq.), Boc_2_O (434 mg, 1.99 mmol, 2 eq.) and DMAP (121 mg, 0.994 mmol, 1 eq.) were dissolved in CH_2_Cl_2_ (50 mL) and stirred for 3 days at rt. The solvent was then removed *in vacuo* at rt to avoid thermal degradation. Purified by flash column chromatography (10% EtOAc:Hexane) to yield N,N’-(*tert*-butylcarbonyl)-6,6’-azidomethyl-indigo, **20** (238 mg, 0.416 mmol, 42%) a pink solid.

**^1^H NMR** (500 MHz, CDCl_3_) δ 8.04 (s, 2H, H***_i_***), 7.77 (dd, *J* = 7.8, 0.6 Hz, 2H, H***_c_***), 7.17 (dt, 2H, H***_d_***), 4.48 (s, 4H, H***_a_***), 1.62 (s, 18H, H***_l_***).

**^13^C NMR** (126 MHz, CDCl_3_) δ 182.9 (C***_j_***), 149.8 (C***_f_***), 149.4 (C***_h_***), 144.0 (C***_b_***), 125.2 (C***_g_***), 124.5 (C***_c_***), 123.7 (C***_d_***), 122.6 (C***_e_***), 116.0 (C***_i_***), 84.9 (C***_k_***), 54.8 (C***_a_***), 28.1 (C***_l_***).

**HR-ESI-MS**: *m/z* = 595.2008 [M+Na]^+^ and *m/z* = 595.2013 [M+Na]^+^, calc. 595.2024 for C_28_H_28_N_8_NaO_6_. Two were masses observed and attributed to the *E* and *Z* isomers of **20**

**FTIR** (neat): ν_max_ 2921.77 (w), 2850.92 (w), 2102.56 (m), 1730.81 (m), 1677.53 (m), 1609.71 (m), 1248.69 (m), 1141.70 (s), 1110.90 (s), 1012.06 (m)

**R.f.**: 0.63 (30% EtOAc:Hexane). Pink spot visible to eye

**M.p.**: 94.0 – 103.8 °C

Figure S22 - ^1^H NMR (CDCl_3_, 500 MHz) of **20**

Figure S23 - JMOD NMR (CDCl_3_, 126 MHz) of **20**

Figure S24 - HMBC NMR (CDCl_3_) of **20**

Figure S25 - HSQC NMR (CDCl_3_) of **20**

Figure S26 - COSY NMR (CDCl_3_) of **20**

Figure S27 - FTIR (neat) of **20**

## 1.2 Synthesis of Dimethyl pyridine-2,6-dicarboxylate, 6

Methanol (300 mL) was cooled to 0 °C and stirred. SOCl_2_ (9.6 mL, 15.7 g, 132.3 mmol, 2.15 eq.) was added dropwise. Dipicolinic acid (**5**) (10.268 g, 61.4 mmol, 1 eq.) was then added slowly. The reaction mixture was then headed to reflux for 5 hours. The solvent was removed *in vacuo* to yield pure **6** as a white crystalline powder (11.6 g, 59.6 mmol, 97%).

**^1^H NMR** (500 MHz, CDCl_3_) δ 8.31 (d, *J* = 7.8 Hz, 2H), 8.02 (dd, *J* = 8.0, 7.6 Hz, 1H), 4.02 (s, 6H).

**^13^C NMR** (126 MHz, CDCl_3_) δ 165.2, 148.4, 138.5, 128.2, 53.4.

**HR-ESI-MS**: *m/z* = 196.0608 [M+H]^+^ calc. 196.0604 for C_9_H_10_NO_4_, *m/z* = 218.0436 [M+Na]^+^ calc. 218.0424 for C_9_H_9_NNaO_4_

This matches reported spectroscopic data from the utilised procedure.^[5]^

Figure S28 - ^1^H NMR (CDCl_3_, 500 MHz) of **6**

Figure S29 - JMOD NMR (CDCl_3_, 126 MHz) of **6**

## 1.3 Synthesis of 2,6-Pyridinedimethanol, 7

**6** (10 g, 51.2 mmol, 1 eq.) was dissolved in dry THF (100 mL) under an argon atmosphere and cooled to 0 °C. NaBH_4_ (8.33g, 220 mmol, 4.3 eq.) was added portion wise and the reaction was stirred overnight and allowed to warm to rt.

NaHCO_3_ (sat., 200 mL) was added dropwise then the reaction was heated to 60 °C for 2 hours. The reaction was allowed to cool and concentrated *in vacuo*. The residue was then extracted with CH_2_Cl_2_/MeOH (9:1). The extraction solution was then condensed *in* *vacuo* to give the desired product, **7** (6.28g, 45.1 mmol, 88%), a white powder.

^1^H NMR (500 MHz, CDCl_3_) δ 7.69 (t, *J* = 7.7 Hz, 1H), 7.19 (dt, *J* = 7.8, 0.6 Hz, 2H), 4.76 (s, 4H), 3.47 (s, 2H).

^13^C NMR (126 MHz, CDCl_3_) δ 158.6, 137.6, 119.4, 64.4.

HR-ESI-MS: *m/z* = 140.0709 [M+H]^+^ calc. 140.0706 for C_7_H_10_NO_2_

This matches reported spectroscopic data from the utilised procedure.^[5]^

Figure S30 - ^1^H NMR (CDCl_3_, 500 MHz) of **7**

Figure S31 - JMOD NMR (CDCl_3_, 126 MHz) of **7**

## 1.4 Synthesis of 2,6-Bis(bromomethyl)pyridine, 8

To a flask of **7** (1.238g, 8.90 mmol, 1eq.) under an argon atmosphere was added HBr (33%, 16.2 mL, 98.8 mmol, 11.1 eq.) slowly and then heated to 125 °C and left stirring overnight. The reaction was then diluted with H_2_O (50 mL) and NaHCO_3_ sln. (sat.) was added until the mixture was pH 8 and extracted with CH_2_Cl_2_ (3 x 150 mL). The organic layers were combined, dried with MgSO_4_ and concentrated *in vacuo*. The product was purified by flash column chromatography (EtOAc:Hexane 10%) to yield **8** (1.019 g, 3.81 mmol, 43%) a white powder. The monobrominated product, **22,** a blossom pink powder was also isolated from the reaction.

**^1^H NMR** (500 MHz, CDCl_3_) δ 7.71 (t, *J* = 7.7 Hz, 1H), 7.38 (d, *J* = 7.7 Hz, 2H), 4.54 (s, 4H).

**^13^C NMR** (126 MHz, CDCl_3_) δ 156.9, 138.3, 123.0, 33.6.

**HR-MS-ESI**: *m/z* = 263.9018 [M+H]^+^ calc. 263.9018 for C_7_H_8_Br_2_N

Figure S32 - ^1^H NMR (CDCl_3_, 500 MHz) of **8**

Figure S33 - JMOD NMR (CDCl_3_, 126 MHz) of **8**

**^1^H NMR** (500 MHz, CDCl_3_) δ 7.69 (t, *J* = 7.7 Hz, 1H), 7.35 (d, *J* = 0.8 Hz, 1H), 7.17 (d, *J* = 0.9 Hz, 1H), 4.76 (s, 2H), 4.55 (s, 2H), 3.69 (s, 1H).

**^13^C NMR** (126 MHz, CDCl_3_) δ 159.1, 155.9, 137.9, 122.2, 119.9, 64.1, 33.6.

**HR-MS-ESI**: m/z = 201.9863 [M+H]^+^ calc. 201.9862 for C_7_H_9_BrNO

These both match reported spectroscopic data from the utilised procedure.^[6]^

Figure S34 - ^1^H NMR (CDCl_3_, 500 MHz) of **22**

Figure S35 - JMOD NMR (CDCl_3_, 126 MHz) of **22**

## 1.5 Synthesis of 2-(4-(2-hydroxyethoxy)phenoxy)ethyl 4-tosylate, 10

2,2'-(1,4-Phenylenebis(oxy))diethanol, **9** (6.00 g, 30.2 mmol, 1 eq.), *p*TsCl (6.06 g, 31.8 mmol, 1.05 eq.) and DMAP (10 mg, 0.08 mmol, cat.) were dissolved in dry THF (150 mL) under an argon atmosphere. NEt_3_ (5.5 mL, 3.99 g, 39.3 mmol, 1.3 eq.) was then added and the reaction mixture stirred overnight at room temperature. The resulting suspension was then filtered, and the solvent of the filtrate was removed *in vacuo* to give the unpurified material.

Purified via flash column chromatography (EtOAc in Hexanes 20% 🡪 40%) to yield **10** (4.45 g, 12.6 mmol, 42 %) a colourless oil.

**^1^H NMR** (500 MHz, CDCl_3_) δ 7.81 (d, *J* = 8.3 Hz, 2H), 7.34 (d, *J* = 8.0 Hz, 2H), 6.82 (d, *J* = 9.2 Hz, 2H), 6.73 (d, *J* = 9.2 Hz, 2H), 4.36 – 4.32 (m, 2H), 4.12 – 4.08 (m, 2H), 4.04 – 4.00 (m, 2H), 3.95 – 3.91 (m, 2H), 2.45 (s, 3H).

**^13^C NMR** (126 MHz, CDCl_3_) δ 153.4, 152.6, 145.1, 133.0, 130.0, 128.1, 115.9, 115.7, 69.9, 68.4, 66.4, 61.7, 21.8.

**HR-ESI-MS**: m/z = 353.1070 [M+H]^+^ calc. 350.1057 for C_17_H_20_NO_5_S, 375.0886 [M+Na]^+^, calc. 372.0876 for C_17_H_19_NNaO_5_S

This matches reported spectroscopic data from the utilised procedure.^[7]^

Figure S36 - ^1^H NMR (CDCl_3_, 500 MHz) of **10**

Figure S37 - JMOD NMR (CDCl_3_, 126 MHz) of **10**

## 1.6 Synthesis of 2-(4-(2-azidoethoxy)phenoxy)ethanol, 11

**10** (5 g, 14.2. mmol, 1 eq.) was dissolved in degassed DMF (24 mL) under an argon atmosphere. NaN_3_ (0.922 g, 14.2 mmol, 1 eq.) was added and the reaction stirred for 48 hrs at rt. The solvent was then removed *in vacuo* and the residue re-dissolved in EtOAc (200 mL). The mixture was washed with H_2_O (3 x 200 mL), LiCl sln. (5% w/w, 200 mL) and brine (200 mL) then dried with MgSO_4_ and concentrated *in vacuo*. The product was purified via flash column chromatography (30% EtOAc in Hexanes) to afford **11** (2.109 g, 9.45 mmol, 67%), a colourless oil.

**^1^H NMR** (500 MHz, CDCl_3_) δ 6.87 (s, 4H), 4.13 – 4.09 (m, 2H), 4.06 – 4.03 (m, 2H), 3.97 – 3.92 (m, 2H), 3.57 (t, *J* = 5.1 Hz, 2H), 2.02 (t, *J* = 6.1 Hz, 1H).

**^13^C NMR** (126 MHz, CDCl_3_) δ 152.9, 115.9, 115.8, 70.0, 67.8, 61.7, 50.4.

**HR-EI-MS**: *m/z* = 109.0285 [M-C_4_H_8_ON_3_]^+•^ (100) calc. 109.0284 for C_6_H_5_O_2_; *m/z* = 233.0952 [M]^+•^ (32) calc. 233.0951 for C_10_H_13_O_3_N_3_

This matches reported spectroscopic data from the utilised procedure.^[7]^

Figure S38 - ^1^H NMR (CDCl_3_, 500 MHz) of **11**

Figure S39 - JMOD NMR (CDCl_3_, 126 MHz) of **11**

## 1.7 Synthesis of 2,6-bis((2-(4-(2-azidoethoxy)phenoxy)ethoxy)methyl)pyridine, 12

**11** (1.712 g, 7.67 mmol, 2 eq.) was dissolved in dry THF (40 mL) under an argon atmosphere. NaH (0.339 g, 8.47 mmol, 2.2 eq.) was added at 0 °C and stirred for 20 minutes at rt. **8** (1.091 g, 3.85 mmol, 1 eq.) was dissolved in dry THF (10 mL) under an argon atmosphere and added to the reaction mixture which was stirred at 1 hour at rt and the heated to 50 °C for 48 hours. The reaction mixture was concentrated *in vacuo*, re-dissolved in EtOAc (200 mL) and partitioned with H_2_O (200 mL). The aqueous phase was then collected and extracted with EtOAc (2 x 200 mL). The organic phases were then combined and dried with MgSO_4_ and the solvent was removed *in vacuo.* The product was purified by flash column chromatography (40% EtOAc in Hexanes) to afford **12** (1.298 g, 2.36 mmol, 61%), a white solid.

**^1^H NMR** (500 MHz, CDCl_3_) δ 7.71 (t, *J* = 7.7 Hz, 1H), 7.39 (dt, *J* = 7.8, 0.6 Hz, 2H), 6.91 – 6.82 (m, 8H), 4.73 (s, 4H), 4.17 – 4.12 (m, 4H), 4.13 – 4.08 (m, 4H), 3.94 – 3.88 (m, 4H), 3.59 – 3.54 (m, 4H).

**^13^C NMR** (126 MHz, CDCl_3_) δ 157.9, 153.6, 152.7, 137.5, 120.2, 115.8, 115.8, 74.4, 69.7, 68.2, 67.8, 50.4.

**HR-ESI-MS**: m/z = 550.2413 [M+H]^+^ calc. 550.2409 for C_27_H_32_N_7_O_6_, 572.2231 [M+Na]^+^ calc. 572.2228 for C_27_H_31_N_7_NaO_6_

This matches reported spectroscopic data from the utilised procedure.^[7]^

Figure S40 - ^1^H NMR (CDCl_3_, 500 MHz) of **12**

Figure S41 - JMOD NMR (CDCl_3_, 126 MHz) of **12**

## 1.8 Synthesis of 2,2'-((((((pyridine-2,6-diylbis(methylene))bis(oxy))bis(ethane-2,1-diyl))bis(oxy))bis(4,1-phenylene))bis(oxy))bis(ethan-1-amine), 13

**12** (0.608 g, 1.11 mmol, 1 eq.) and Pd/C (10 wt. %, 0.608g, 5.71 mmol, 5.16 eq.) were dissolved in dry MeOH (58 mL) under an argon atmosphere. H_2_NNH_2_·H_2_O (55%, 0.98 mL, 1.01 g, 11.1 mmol, 10 eq.) was added to the reaction mixture which was then heated to reflux and stirred overnight under an argon atmosphere.

The reaction mixture was cooled to rt and filtered through a celite plug. Solvent was removed *in vacuo* to give **13** (0.401 g, 0.805 mmol, 73%) a white solid which was taken forward without any further purification.

**^1^H NMR** (500 MHz, CDCl_3_) δ 7.58 (t, *J* = 7.7 Hz, 1H), 7.39 (d, *J* = 8.1 Hz, 2H), 6.90 – 6.78 (m, 8H), 4.72 (d, *J* = 8.4 Hz, 4H), 4.34 – 4.25 (m, 4H), 4.16 – 4.10 (m, 4H), 4.00 – 3.93 (m, 4H), 3.93 – 3.88 (m, 4H).

**13C NMR** (126 MHz, CDCl3) δ 157.9, 153.3, 153.3, 137.5, 120.3, 115.8, 115.5, 74.3, 69.6, 68.2, 29.9.

**HR-ESI-MS**: m/z = 498.2604 [M+H]^+^ calc. 498.2599 for C_27_H_36_N_3_O_6_, 520.2422 [M+Na]^+^ calc. 520.2418 for C_27_H_35_N_3_NaO_6_

This matches reported spectroscopic data from the utilised procedure.^[7]^

Figure S42 - ^1^H NMR (CDCl_3_, 500 MHz) of **13**

Figure S43 - JMOD NMR (CDCl_3_, 126 MHz) of **13**

## 1.9 Synthesis of 3,6,8,18,20,23-hexaoxa-11,15-diaza-1(2,6)-pyridina-7,19(1,4),13(1,3)-tribenzenacyclotetracosaphane-12,14-dione, 14

**13** (0.486 g, 0.977 mmol, 1 eq.) and triethylamine (0.680 mL, 494 mg, 4.88 mmol, 5 eq.) were dissolved in dry CH_2_Cl_2_ (185 mL) under an argon atmosphere. Isophthaloyl dichloride (0.198 mg, 0.977 mmol, 1 eq.) was dissolved in dry CH_2_Cl_2_ (15 mL) under an argon atmosphere and added to the reaction mixture dropwise over 2 minutes. The mixture was left to stir overnight at room temperature. The reaction mixture was then washed sequentially with citric acid (10%, 200 mL), NaHCO_3_ sln. (sat., 200 mL) and brine (200 mL). The organic phase was then dried with MgSO_4_ and the solvent was removed *in vacuo*. The product was purified by flash column chromatography (2% MeOH in CH_2_Cl_2_ 🡪 5% MeOH in CH_2_Cl_2_) to yield **14** (0.1950 g, 310.7 μmol, 32 %), a white powder.

**^1^H NMR** (500 MHz, DMSO-*d_6_*) δ 8.73 (t, *J* = 5.4 Hz, 2H, H***_m_***), 8.27 (t, *J* = 1.7 Hz, 1H, Ho), 7.95 (dd, *J* = 7.7, 1.8 Hz, 2H, H***_q_***), 7.72 (t, *J* = 7.7 Hz, 1H, H***_a_***), 7.55 (t, *J* = 7.9 Hz, 1H, H***_r_***), 7.30 (d, *J* = 7.7 Hz, 2H, H***_b_***), 6.87 – 6.79 (m, 8H, H***_h_*** & H***_i_***), 4.56 (s, 4H, H***_d_***), 4.09 – 4.04 (m, 8H, H***_f_*** & H***_k_***), 3.84 – 3.78 (m, 4H, H***_e_***), 3.61 (q, *J* = 5.4 Hz, 4H, H***_l_***).

**^13^C NMR** (126 MHz, DMSO-*d_6_*) δ 166.2 (C***_n_***), 157.4 (C***_c_***), 152.6 (C***_g_***), 152.5 (C***_j_***), 137.3 (C***_a_***), 134.5 (C***_p_***), 129.9 (C***_q_***), 128.4 (C***_r_***), 126.0 (C***_o_***), 120.5 (C***_b_***), 115.5 (C***_j_***), 115.5 (C***_g_***), 73.2 (C***_d_***), 68.9 (C***_e_***), 67.6 (C***_f_***), 66.6 (C***_k_***).

**HR-ESI-MS**: *m/z* = 628.2667 [M+H]^+^ calc. 628.2653 for C_35_H_38_N_3_O_8_, *m/z* = 650.2469 [M+Na]^+^ calc. 650.2473 for C_35_H_37_N_3_NaO_8_

**FTIR (neat)**: 3396.21 (w), 3362.37 (w), 2866,83 (w), 1669.32 (s), 1540.04 (s), 1508.10 (s), 1222,47 (s), 1105.58 (s), 821.57 (s), 722.27 (m), 602.39 (m)

**R.f.**: 0.64 (6% MeOH in DCM), visualised by UV

**M.p.**: Decomposes at 219.2 °C

Figure S44 - ^1^H NMR (DMSO-*d_6_*, 500 MHz) of **14**

Figure S45 - JMOD NMR (DMSO-*d_6_*, 126 MHz) of **14**

Figure S46 - HMBC NMR (DMSO-*d_6_*) of **14**

Figure S47 - HSQC NMR (DMSO-*d_6_*) of **14**

Figure S48 - COSY NMR (DMSO-*d_6_*) of **14**

Figure S49 - FTIR (neat) of **14**

## 1.10 Synthesis of Bis(4-(tert-butyl)phenyl)(4-isopropylphenyl)methanol, 2

In an oven-dried flask under an argon atmosphere, 1-bromo-4-(tert-butyl)benzene, **1** (2.50 g, 2.03 mL, 3.30 eq., 11.7 mmol) was dissolved in dry THF (23.0 mL) and cooled to -78 °C. Butyllithium (4.69 mL, 2.50 M in hexane, 3.3 eq., 11.7 mmol) was added over the course of 15 mins and then allowed to stir for 45 min. Diethyl carbonate (0.420 g, 0.431 mL, 1.00 eq., 3.56 mmol) was then added dropwise and the mixture allowed to warm to 25 °C and stirred for 3 hours. The reaction was then quenched with H_2_O (50 mL) and the THF removed *in vacuo*. The remaining aqueous mixture was washed with Et_2_O (3 x 50 mL). The combined organic phases were then washed with NaHCO_3_ sln. (sat., 150 mL ) and brine (150 mL). The organic phases were then dried with MgSO_4_ and the solvent was removed *in vacuo*. The product was purified by column chromatography (20% CH_2_Cl_2_:Hexane) to yield the desired product, **2** (0.882 g, 2.06 mmol, 58 %) a white powder.

**^1^H NMR** (500 MHz, CDCl_3_) δ 7.33 (d, *J* = 8.4 Hz, 6H), 7.20 (d, *J* = 8.3 Hz, 6H), 2.74 (s, 1H), 1.32 (s, 27H).

**^13^C NMR** (126 MHz, CDCl_3_) δ 150.0, 144.3, 127.7, 124.8, 81.7, 34.6, 31.5.

**HR-EI-MS**: *m/z* = 161.0962 [M - 2Ph-*t*Bu]^+•^ (100) calc. 161.0961 for C_11_H_13_O; *m/z* = 295.2059 [M – Ph-*t*Bu] ^+•^ (78) calc. 295.2056 for C_21_H_27_O; *m/z* = 411.3040 [M – OH] ^+•^ (30) calc. 411.3040 for C_31_H_39_; *m/z* = 428.3080 [M]^+•^ (1.5) calc. 428.3074 for C_31_H_40_O

This matches reported spectroscopic data from the utilised procedure.^[7]^

Figure S50 - ^1^H NMR (CDCl_3_, 500 MHz) of **2**

Figure S51 - JMOD NMR (CDCl_3_, 126 MHz) of **2**

## 1.11 Synthesis of 4-(tris(4-(tert-butyl)phenyl)methyl)phenol, 3

**2** (0.800 g, 1.87 mmol, 1 eq.) was dissolved in phenol (7.90 g, 84.0 mmol, 45 eq.) under argon atmosphere at 110 °C. Conc. sulfuric acid (31.4 μL, 0.560 mmol 0.3 eq.) added and the reaction for 20 hours. The reaction was then quenched with NaOH sln. (1 M, 50 mL) and stirred vigorously for 15 mins. The suspension was then filtered and washed with NaOH (1 M, 200 mL) and then with HCl (1 M, 200 mL) to yield **3** (0.773 g, 1.80 mmol, 97 %), a white powder.

**^1^H NMR** (500 MHz, CDCl_3_) δ 7.23 (d, *J* = 8.9 Hz, 6H), 7.07 (d, *J* = 8.8 Hz, 6H), 7.04 (d, *J* = 8.8 Hz, 2H), 6.70 (d, *J* = 8.9 Hz, 2H), 4.62 (s, 1H), 1.30 (s, 27H).

**^13^C NMR** (126 MHz, CDCl_3_) δ 153.4, 148.5, 144.3, 140.0, 132.6, 130.9, 124.2, 114.1, 63.2, 34.4, 31.5.

**LR-EI-MS**: *m/z* = 504.4 [M]^•+^ calc. 504.3 for C_37_H_44_O

This matches reported spectroscopic data from the utilised procedure.^[7]^

Figure S52 - ^1^H NMR (CDCl_3_, 500 MHz) of **3**

Figure S53 - JMOD NMR (CDCl_3_, 126 MHz) of **3**

## 1.12 Synthesis of 4,4',4''-((4-(prop-2-yn-1-yloxy)phenyl)methanetriyl)tris(tert-butylbenzene), 4

**3** (0.954 g, 1.89 mmol, 1 eq.) and 3-bromoprop-1-yne (0.305 mL, 80 wt% in PhMe, 2.84 mmol, 1.5 eq.) were dissolved in DMF (20.0 mL). Potassium carbonate (1.31 g, 9.45 mmol, 5 eq.) was added and the reaction was stirred at 80 °C for 16 hours. The reaction mixture was then diluted with EtOAc (250 mL), washed with water (250 mL) and brine (5 x 250 mL). The organic layer was then dried with MgSO_4_ and the solvent was removed *in vacuo*. The residue was purified by flash column chromatography (20% CH_2_Cl_2_ in hexanes) to afford the desired material **4** (0.967 g, 1.78 mmol, 94 %), a white solid.

**^1^H NMR** (500 MHz, CDCl_3_) δ 7.23 (d, *J* = 8.7 Hz, 6H), 7.11 (d, *J* = 9.2 Hz, 2H), 7.07 (d, *J* = 8.9 Hz, 6H), 6.84 (d, *J* = 9.0 Hz, 2H), 4.66 (d, *J* = 2.4 Hz, 2H), 2.51 (t, *J* = 2.4 Hz, 1H), 1.30 (s, 27H).

**^13^C NMR** (126 MHz, CDCl_3_) δ 155.6, 148.5, 144.2, 140.7, 132.4, 130.9, 124.2, 113.5, 79.0, 75.5, 63.2, 55.9, 34.4, 31.5.

**LR-EI-MS**: 542.5 [M]^+•^ calc. 542.4 for C_40_H_46_O

This matches reported spectroscopic data from the utilised procedure.^[7]^

Figure S54 - ^1^H NMR (CDCl_3_, 500 MHz) of **4**

Figure S55 - JMOD NMR (CDCl_3_, 126 MHz) of **4**

## 1.13 Synthesis of *N,N’*-Methyl Indigo [2]rotaxane, Me-Rot

**14** (21.8 mg, 34.7 μmol, 1 eq.) and Cu(CH_3_CN)_4_PF_6_ (12.9 mg, 34.7 μmol, 1 eq.) were dissolved in dry, sparged CHCl_3_ (1 mL) under an argon atmosphere and stirred at rt.

**19** (80.2 mg, 140 μmol, 2.93 eq.) and **4** (152 mg, 281 μmol, 5.87 eq.) were dissolved in dry, sparged CHCl_3_ (1 mL) under an argon atmosphere and added to the stirring solution. The mixture was then stirred at rt overnight.

The solution was transferred to a separating funnel, diluted with CHCl_3_ (100 mL) and partitioned with EDTA-NH_3_ sln. (sat., 100 mL). The organic phase was collected and the aqueous phase was extracted with CH_2_Cl_2_ (4 x 100 mL). The combined organic phases were then washed with brine (500 mL), dried with MgSO_4_ and the solvent was removed *in vacuo*.

The product was purified by flash column chromatography (Column 1: 20% → 100% EtOAc in hexanes, then 0% → 20% MeOH in CH_2_Cl_2_. Column 2: 20% → 50% Acetone:CH_2_Cl_2_. Prep TLC: 20% MeOH:Toluene) to yield **Me-Rot** (11 mg, 5.20 μmol, 14%), a blue powder.

**^1^H NMR** (500 MHz, CDCl_3_) δ 8.75 (t, *J* = 1.8 Hz, 1H, H***_O_***), 8.22 (dd, *J* = 7.8, 1.6 Hz, 2H, H***_Q_***), 7.90 (s, 2H, H***_n_***), 7.61 (t, *J* = 7.8 Hz, 1H, H***_R_***), 7.53 (t, *J* = 5.3 Hz, 2H, H***_M_***), 7.32 (t, *J* = 7.7 Hz, 1H, H***_A_***), 7.28 (d, *J* = 7.8 Hz, 2H, H***_r_***), 7.22 – 7.19 (m, 12H, H***_d_***), 7.15 – 7.11 (m, 4H, H***_i_***), 7.08 – 7.05 (m, 12H, H***_e_***), 6.96 (d, *J* = 7.8 Hz, 2H, H***_B_***), 6.93 – 6.87 (m, 4H, H***_j_***), 6.51 (d, *J* = 7.9 Hz, 2H, H***_q_***), 6.47 (s, 2H, H***_w_***), 6.20 – 6.11 (m, 8H, H***_H_*** & H***_I_***), 5.25 (s, 4H, H***_o_***), 5.21 (s, 4H, H***_l_***), 4.58 (s, 4H, H***_D_***), 3.93 (d, *J* = 1.7 Hz, 8H, H***_F_*** & H***_K_***), 3.67 (t, *J* = 4.4 Hz, 4H, H***_E_***), 3.48 (q, *J* = 5.0 Hz, 4H, H***_L_***), 3.28 (s, 6H, H***_x_***), 1.29 (s, 54H, H***_a_***).

**^13^C NMR** (126 MHz, CDCl_3_) δ 184.9 (C***_t_***), 166.4 (C***_N_***), 156.3 (C***_C_***), 156.2 (C***_h_***), 152.5 (C***_s_***), 152.3 (C***_G_***), 151.7 (C***_J_***), 148.4 (C***_c_***), 144.6 (C***_m_***), 144.0 (C***_f_***), 143.1 (C***_v_***), 140.2 (C***_k_***), 137.5 (C***_A_***), 133.9 (C***_P_***), 132.4 (C***_i_***), 132.0 (C***_Q_***), 130.7 (C***_e_***), 129.5 (C***_R_***), 126.4 (C***_u_***), 124.1 (C***_r_***), 124.1 (C***_d_***), 123.9 (C***_n_***), 123.5 (C***_O_***), 122.6 (C***_B_***), 120.6 (C***_p_***), 120.2 (C***_q_***), 114.6 (C***_H_***), 114.0 (C***_I_***), 113.2, (C***_j_***) 109.8 (C***_w_***), 74.6 (C***_D_***), 69.9 (C***_F_***), 67.4 (C***_K_***), 66.8 (C***_E_***), 63.0 (C***_g_***), 61.9 (C***_l_***), 53.7 (C***_o_***), 39.8 (C***_L_***), 36.4 (C***_x_***), 34.3 (C***_b_***), 31.4 (C***_a_***).

**ESI-TOF**: m/z = 2135.2 [M+Na]^+^ calc. 2135.1 for C_135_H_145_N_11_O_12_Na

**FTIR (neat)**: 3326.35 (br w), 2956.96 (w), 2867.94 (w), 1642.63 (m), 1617.49 (m), 1534.98 (m), 1505.70 (s), 1458.03 (m), 1391.19 (w), 1362.78 (w), 1201.77 (s), 1089.85 (s), 1065.71 (s), 1015.76 (s), 921.40 (w), 822.22 (s), 794.32 (m), 724.83 (m), 701.86 (m), 636.64 (w), 580.72 (m)

**R.f.**: 0 (40% EtOAc:Hexane), 0.59 & 0.69 (5% MeOH:CH_2_Cl_2_; *E* & *Z* Isomers observed), 0.5 (20% MeOH:Toluene)

**M.p.**: 152.7 – 167.3 °C

Figure S56 - ^1^H NMR (CDCl_3_, 500 MHz) of **Me-Rot**

Figure S57 - JMOD NMR (CDCl_3_, 126 MHz) of **Me-Rot**

Figure S58 - HMBC NMR (CDCl_3_) of **Me-Rot**

Figure S59 - HSQC NMR (CDCl_3_) of **Me-Rot**

Figure S60 - COSY NMR (CDCl_3_) of **Me-Rot**

Figure S61 - NOESY NMR (CDCl_3_) of **Me-Rot**

Figure S62 - Isotope pattern for **Me-Rot**, C_135_H_145_N_11_O_12_Na.

Figure S63 - FTIR (neat) of **Me-Rot**

## 1.14 Synthesis of *N,N’*-Boc Indigo [2]rotaxane, Boc-Rot

**14** (30.0 mg, 47.8 μmol, 1 eq.) and Cu(CH_3_CN)_4_PF_6_ (17.8 mg, 47.8 μmol, 1 eq.) were dissolved in dry, sparged CH_2_Cl_2_ (1.50 mL) under an argon atmosphere and stirred at rt. **20** (80.2 mg, 140 μmol, 2.93 eq.) and **4** (152 mg, 281 μmol, 5.87 eq.) were dissolved in dry, sparged CH_2_Cl_2_ (1.50 mL) under an argon atmosphere and added to the stirring solution. The mixture was then stirred at rt overnight. The solution was transferred to a separating funnel, diluted with CH_2_Cl_2_ (100 mL) and partitioned with EDTA-NH_3_ sln. (sat., 100 mL). The organic phase was collected and the aqueous phase was extracted with CH_2_Cl_2_ (4 x 100 mL). The combined organic phases were then washed with brine (500 mL), dried with MgSO_4_ and concentrated *in vacuo*. The product was purified by flash column chromatography (40% EtOAc in hexanes, then 3% MeOH in CH_2_Cl_2_) to yield **Boc-Rot** (32 mg, 47.8 μmol, 29%) as a pink powder.

Due to steric restriction from the Boc groups, the axle of the [2]rotaxane, **Boc-Rot** is desymmetrised. Consequently, there are multiple NMR signals for what would ordinarily appear to be the same environment.

**^1^H NMR** (500 MHz, CD_3_CN) δ 8.51 (s, 1H, H***_O_***), 8.01 – 7.98 (m, 2H, H***_Q_***), 7.96 (s, 1H, H***_n_***), 7.77 – 7.58 (m, 5H, H***_q_***, H***_r_***, H***_r’_***, H***_n’_***, & H***_A_***), 7.44 (t, *J* = 7.8 Hz, 1H, H***_R_***), 7.30 (s, 2H, H***_M_***), 7.27 – 7.15 (m, 15H, H***_w_***, H***_w’_***, H***_q’_***, H***_d_***, & H***_d’_***), 7.13 – 7.05 (m, 16H, H***_e_***, H***_e’_***, & H***_i_***), 7.03 (d, J = 8.3 Hz, 2H, H***_i’_***), 6.86 (d, *J* = 8.4 Hz, 2H, H***_j_***), 6.67 (d, *J* = 8.4 Hz, 2H, H***_j’_***), 6.29 (s, 8H, H***_H_*** & H***_I_***), 5.69 (s, 2H, H***_l’_***), 5.12 (s, 4H, H***_l_*** & H***_o’_***), 4.89 (s, 2H, H***_l’_***), 4.71 (m, 4H, H***_D_***), 4.07 – 3.29 (m, 16H, H***_E_***, H***_F_***, H***_K_***, & H***_L_***), 1.49 (s, 9H, H***_z_***), 1.39 (s, 9H, H***_z’_***), 1.26 (d, *J* = 3.1 Hz, 54H, H***_a_*** & H***_a’_***).

**^13^C NMR** (126 MHz, CD_3_CN) δ 182.4 (C***_t_***), 182.0 (C***_t’_***), 165.8 (C***_N_***), 155.4 (C***_x_***), 155.1 (C***_x’_***), 151.5 (C***_G_***), 151.4 (C***_J_***), 148.9 (C***_s_***), 148.7 (C***_s’_***), 148.3 (C***_v_***), 147.8 (C***_v’_***), 147.5 (C***_f_*** & C***_f’_***), 147.5 (C***_h_*** & C***_h’_***), 143.5 (C***_c_*** & C***_c’_***), 143.4 (C***_m_***), 143.4 (C***_m’_***), 142.9 (C***_p_***), 142.7 (C***_p’_***), 139.2 (C***_k_*** & C***_k’_***), 133.6 (C***_P_***), 131.0 (C***_i_***), 131.0 (C***_i’_***), 130.0 (C***_Q_***), 129.5 (C***_d_*** & C***_d’_***), 128.0 (C***_R_***), 124.5 (C***_u_***), 124.2 (C***_u’_***), 124.0 (C***_B_***), 124.0 (C***_w_***), 123.7 (C***_C_***), 123.6 (C***_w’_***), 123.5 (C***_e_*** & C***_e’_***), 123.2 (C***_q_***), 123.2 (C***_q’_***), 122.5 (C***_r_***), 121.6 (C***_r’_***), 115.0 (C***_n_***), 114.8 (C***_n’_***), 114.7 (C***_A_***), 114.2 (C***_H_***), 113.9 (C***_I_***), 112.6 (C***_j_***), 112.4 (C***_j’_***), 84.1 (C***_y_***), 84.0 (C***_y’_***), 68.9 (C***_F_***), 67.2 (C***_g_***), 66.8 (C***_K_***), 66.2 (C***_g’_***), 65.6 (C***_E_***), 62.2 (C***_D_***), 60.7 (C***_l_***), 60.2 (C***_l’_***), 52.8 (C***_o_***), 52.4 (C***_o’_***), 38.5 (C***_L_***), 33.3 (C***_b_*** & C***_b’_***), 30.1 (C***_a_*** & C***_a’_***), 26.8 (C***_z_***), 26.7 (C***_z’_***).

**ESI-TOF**: m/z = 1143.1 [M+2H]^2+^ calc. 2286.20 for C_145_H_157_N_11_O_16_

**FTIR (neat)**: 2959.71 (w) 2867.54 (w), 1741.41 (w), 1671.85 (m), 1613.18 (m), 1505.29 (s), 1458.38 (m), 1437.98 (m), 1394.05 (w), 1362.02 (m), 1228.68 (m), 1145.58 (m), 1108.81 (m), 1081.03 (m), 1042.00 (m), 1013.72 (s), 914.37 (w), 820.65 (s), 730.68 (m), 701.58 (m), 637.31 (w), 579.48 (m)

**R.f.**: 0.26 (5% MeOH in CH_2_Cl_2_). Pink spot visible to eye

**M.p.**: 135.6 – 146.8 °C

Figure S64 - ^1^H NMR (CD_3_CN: CDCl_3_ (1:1), 500 MHz) of **Boc-Rot**

Figure S65 - JMOD NMR (CD_3_CN:CDCl_3_ (1:1), 126 MHz) of **Boc-Rot**

Figure S66 - HMBC NMR (CD_3_CN: CDCl_3_ (1:1)) of **Boc-Rot**

Figure S67 - HSQC NMR (CD_3_CN: CDCl_3_ (1:1)) of **Boc-Rot**

Figure S68 - COSY NMR (CD_3_CN: CDCl_3_ (1:1)) of **Boc-Rot**

Figure S69 - NOESY NMR (CD_3_CN: CDCl_3_ (1:1)) of **Boc-Rot**

Figure S70 - Isotope pattern for **Boc-Rot** C_145_H_157_N_11_O_16_

Figure S71 - FTIR (neat) of **Boc-Rot**

## 1.15 Synthesis of di-tert-butyl-3,3'-dioxo-6,6'-bis((4-((4-(tris(4-(tert-butyl)phenyl)methyl)phenoxy)methyl)-1H-1,2,3-triazol-1-yl)methyl)-[2,2'-biindolinylidene]-1,1'-dicarboxylate, Boc-Axle

A suspension of CuI (3 mg, 0.0175 mmol, 0.2 eq.) and TBTA (9 mg, 0.0175 mmol, 2 eq.) in argon sparged, dry THF (2.5 mL) was added a solution of **4** (99 mg, 0.183 mmol, 2.1 eq.) and **20** (50 mg, 0.0873 mmol, 1 eq.) in argon sparged, dry THF (2.5 mL). The reaction mixture was heated to reflux (70 °C) and stirred overnight under an argon atmosphere. The reaction mixture was concentrated *in vacuo*, and the residue was redissolved in EtOAc (40 mL). The solution was washed with H_2_O (3 x 40 mL) and brine (40 mL). The organic phase was dried with MgSO_4­_ and the solvent was removed *in vacuo*. The product was purified by flash column chromatography (30% EtOAc:Hexane → 60% EtOAc:Hexane) to yield **Boc-Axle** (95 mg, 0.0573 mmol, 66%) a pink powder.

**^1^H NMR** (500 MHz, CDCl_3_) δ 7.94 (s, 2H, H***_n_***), 7.75 (dd, *J* = 7.8, 0.6 Hz, 2H, H***_q_***), 7.63 (s, 2H, H***_w_***), 7.25 – 7.20 (m, 12H, H***_d_***), 7.12 – 7.04 (m, 18H, H***_e_***, H***_i_*** & H***_r_***), 6.87 – 6.82 (m, 4H, H***_j_***), 5.63 (s, 4H, H***_l_***), 5.20 – 5.16 (m, 4H, H***_o_***), 1.57 (s, 18H, H***_z_***), 1.29 (s, 54H, H***_a_***).

**^13^C NMR** (126 MHz, CDCl_3_) δ 182.7 (C***_t_***), 156.1 (C***_y_***), 149.6 (C***_h_***), 149.3 (C***_s_***), 148.4 (C***_c_***), 145.3 (C***_v_***), 144.0 (C***_f_***), 142.7 (C***_p_***), 140.3 (C***_k_***), 132.3 (C***_i_***), 130.7 (C***_e_***), 125.0 (C***_u_***), 124.8 (C***_q_***), 124.0 (C***_d_***), 123.6 (C***_r_***), 123.0 (C***_m_***), 122.8 (C***_w_***), 116.0 (C***_n_***), 113.2 (C***_j_***), 85.2 (C***_y_***), 63.0 (C***_g_***), 62.0 (C***_o_***), 54.2 (C***_l_***), 34.3 (C***_b_***), 31.4 (C***_a_***), 28.1 (C***_z_***).

**ESI-TOF**: m/z = 1679.9 [M+Na]^+^ calc. 1679.91 for C_108_H_120_N_8_O_8_Na

**FTIR** (neat): 2977.06 (w), 2931.81 (w), 2096.43 (m), 1734.69 (m), 1674.17 (m), 1609.96 (m), 1432.15 (m), 1241.43 (m), 1141.00 (s), 1109.46 (s), 1074.67 (s), 1011.67 (m), 750.14 (m)

**R.f.**: 0.59 (30% EtOAc:Hexane), pink spot clearly visible

**M.p.**: 90.9 – 96.7 °C

Figure S72 - ^1^H NMR (CDCl_3_, 500 MHz) of **Boc-Axle**

Figure S73 - JMOD NMR (CDCl_3_, 126 MHz) of **Boc-Axle**

Figure S74 - HMBC NMR (CDCl_3_) of **Boc-Axle**

Figure S75 - HSQC NMR (CDCl_3_) of **Boc-Axle**

Figure S76 - COSY NMR (CDCl_3_) of **Boc-Axle**

Figure S77 - NOESY NMR (CDCl_3_) of **Boc-Axle**

Figure S78 - Isotope pattern of **Boc-Axle**, C_108_H_120_N_8_O_8_Na.

Figure S79 - FTIR (neat) of **Boc-Axle**

## 1.16 Synthesis of 6,6'-bis((4-((4-(tris(4-(tert-butyl)phenyl)methyl)phenoxy)methyl)-1H-1,2,3-triazol-1-yl)methyl)-[2,2'-biindolinylidene]-3,3'-dione, 21

**Boc-Axle** (100 mg, 0.0603 mmol, 1 eq.) was dissolved in CH_2_Cl_2_:TFA (1:1, 25 mL) and stirred for 90 mins. The solvent was removed *in vacuo* and re-dissolved in CH_2_Cl_2_ (25 mL). K_2_CO_3_ (~5 g) was suspended in the solution and stirred for 30 mins to remove any remaining TFA. The suspension was filtered and the solvent was removed *in vacuo* to yield **21** (83 mg, 0.0573 mmol, 95%) as a blue powder.

**^1^H NMR** (500 MHz, CDCl_3_) δ 8.94 (s, 2H, H***_x_***), 7.71 (d, *J* = 7.9 Hz, 2H, H***_r_***), 7.63 (s, 2H, H***_n_***), 7.24 – 7.20 (m, 12H, H***_d_***), 7.11 – 7.08 (m, 4H, H***_i_***), 7.08 – 7.04 (m, 12H, H***_e_***), 6.89 (s, 2H, H***_w_***), 6.87 (dd, *J* = 7.9, 1.4 Hz, 2H, H***_q_***), 6.85 – 6.82 (m, 4H, H***_j_***), 5.57 (s, 4H, H***_l_***), 5.19 (s, 4H, H***_o_***), 1.29 (s, 54H, H***_a_***).

**^13^C NMR** (126 MHz, CDCl_3_) δ 187.9 (C***_t_***), 156.2 (C***_h_***), 152.2 (C***_s_***), 148.5 (C***_c_***), 145.4 (C***_m_***), 144.12 (C***_f_***), 143.2 (C***_p_***), 140.5 (C***_k_***), 132.5 (C***_i_***), 130.9 (C***_e_***), 125.5 (C***_r_***), 124.2 (C***_d_***), 123.0 (C***_n_***), 121.7 (C***_v_***), 120.5 (C***_q_***), 120.3 (C***_u_***), 113.3 (C***_j_***), 111.5 (C***_w_***), 63.2 (C***_g_***), 62.2 (C***_o_***), 54.2 (C***_l_***), 34.4 (C***_b_***), 31.5 (C***_a_***).

**MALDI-TOF**: m/z = 1479.7982 [M+Na]^+^ calc. 1479.8073 for C_98_H_104_N_8_O_8_Na

**FTIR (neat)**: 3363.44 (br w), 2957.57 (w), 2866.19 (w), 1675.09 (m), 1646.78 (s), 1617.92 (s), 1504.10 (w), 1444.96 (m), 1393.06 (m), 1362.32 (m), 1269.60 (m), 1247.59 (m), 1190.71 (s), 1132.71 (s), 1074.26 (m), 1049.23 (m), 1005.83 (m), 822.09 (s), 803.69 (m), 769.04 (m), 723.22 (m), 699.87 (s), 661.32 (m), 595.74 (m), 578.88 (m)

**R.f.**: 0.36 (2% MeOH in CH_2_Cl_2_). Blue spot visible to the eye

**M.p.**: Decomposed at 245.2 °C

Figure S80 - ^1^H NMR (CDCl_3_, 500 MHz) of **21**

Figure S81 - JMOD NMR (CDCl_3_, 126 MHz) of **21**

Figure S82 - HMBC NMR (CDCl_3_) of **21**

Figure S83 - HSQC NMR (CDCl_3_) of **21**

Figure S84 - COSY NMR (CDCl_3_) of **21**

Figure S85 - NOESY NMR (CDCl_3_) of **21**

Figure S86 - Isotope pattern of **21**, C_98_H_104_N_8_O_8_Na.

Figure S87 - FTIR (neat) of **21**

## 1.17 Synthesis of 1,1'-dimethyl-6,6'-bis((4-((4-(tris(4-(tert-butyl)phenyl)methyl)phenoxy)methyl)-1H-1,2,3-triazol-1-yl)methyl)-[2,2'-biindolinylidene]-3,3'-dione, Me-Axle

**21** (15 mg, 0.0103 mmol, 1 eq.) was dissolved in DMF (2 mL) under ambient conditions. Cs_2_CO_3_ (7 mg, 0.0216 mmol, 2.1 eq.) and MeI (2.6 μL, 6 mg, 0.0423 mmol, 4.1 eq.) were added to the solution and stirred overnight at rt. The reaction mixture was diluted with EtOAc (25 mL) and washed with brine (5 x 25 mL). The organic phase was collected, dried with MgSO_4_ and the solvent was removed *in vacuo* to yield **Me-Axle** (15 mg, 0.0101 mmol, 98%) a forest green powder.

**^1^H NMR** (500 MHz, CDCl_3_) δ 7.70 (d, *J* = 8.0 Hz, 2H, H***_r_***), 7.62 (s, 2H, H***_n_***), 7.23 – 7.21 (m, 12H, H***_d_***), 7.13 – 7.08 (m, 4H, H***_i_***), 7.07 – 7.05 (m, 12H, H***_e_***), 6.97 – 6.92 (m, 4H, H***_j_***), 6.85 – 6.82 (m, 4H, H***_q_*** & H***_w_***), 5.61 (s, 4H, H***_l_***), 5.19 (s, 4H, H***_o_***), 3.51 (s, 6H, H***_x_***), 1.29 (s, 54H, H***_a_***).

**^13^C NMR** (126 MHz, CDCl_3_) δ 184.2 (C***_t_***), 156.0 (C***_h_***), 153.3 (C***_s_***), 148.4 (C***_f_***), 145.2 (C***_m_***), 144.0 (C***_c_***), 141.8 (C***_v_***), 140.3(C***_k_***), 132.3(C***_i_***), 130.7 (C***_e_***), 126.4 (C***_u_***), 124.8 (C***_r_***), 124.0 (C***_d_***), 122.7 (C***_n_***), 121.8 (C***_p_***), 120.9 (C***_q_***), 113.2 (C***_j_***), 110.0 (C***_w_***), 63.0 (C***_g_***), 62.0 (C***_o_***), 54.3 (C***_l_***), 36.7 (C***_x_***), 34.3 (C***_b_***), 31.4 (C***_a_***).

**MALDI-TOF** : m/z = 1507.8356 [M+Na]^+^ calc. 1507.8386 for C_100_H_108_N_8_O_8_Na

**FTIR (neat)**: 2957.75 (w), 2923.14 (w), 2851.98 (w), 1645.07 (w), 1615.41 (m), 1504.08 (m), 1459.30 (m), 1393.23 (w), 1362.04 (w), 1244.71 (w), 1183.59 (m), 1088.51 (s), 1011.38 (s), 821.40 (s), 769.91 (m), 735.10 (w), 706.37 (w), 580.57 (s)

**R.f.**: 0.09 (50% EtOAc in Hexanes). Green spot visible by eye.

**M.p.**: 155.4 – 157.1 °C

Figure S88 - ^1^H NMR (CDCl_3_, 500 MHz) of **Me-Axle**

Figure S89 - JMOD NMR (CDCl_3_, 126 MHz) of **Me-Axle**

Figure S90 - HMBC NMR (CDCl_3_) of **Me-Axle**

Figure S91 - HSQC NMR (CDCl_3_) of **Me-Axle**

Figure S92 - COSY NMR (CDCl_3_) of **Me-Axle**

Figure S93 - NOESY NMR (CDCl_3_) of **Me-Axle**

Figure S94 - Isotope pattern of **Me-Axle**, C_100_H_108_N_8_O_8_Na.

Figure S95 - FTIR (neat) of **Me-Axle**

## 1.18 Synthesis of N^1^,N^3^-bis(2-phenoxyethyl)isophthalamide

Isopthaloyl chloride (406 mg, 2 mmol, 1 eq.) was dissolved in anhydrous CH_2_Cl_2_ (15 mL) under an argon atmosphere. 2-phenoxyethanamine (**#**) (686 mg, 5 mmol, 2.5 eq.) and NEt_3_ (607 mg, 6 mmol, 3 eq.) were added to the reaction and stirred overnight at rt. The emergence of a white precipitate could be seen and was collected by filtration. The precipitate was washed with water and then cold ethanol, before being dried *in vacuo*, yielding the half-macrocycle, **HM** as a white powder (275 mg, 0.680 mmol, 34%).

**^1^H NMR** (400 MHz, DMSO) δ 8.82 (t, *J* = 5.5 Hz, 2H, H***_f_***), 8.37 (t, *J* = 1.6 Hz, 1H, H***_d_***), 8.00 (dd, *J* = 7.8, 1.8 Hz, 2H, H***_b_***), 7.56 (t, *J* = 7.8 Hz, 1H, H***_a_***), 7.33 – 7.24 (m, 4H, H***_k_***), 7.01 – 6.88 (m, 6H, H***_j_*** & H***_l_***), 4.13 (t, *J* = 5.9 Hz, 4H, H***_h_***), 3.66 (q, *J* = 5.8 Hz, 4H, H***_g_***).

**^13^C NMR** (101 MHz, DMSO) δ 166.1 (C***_e_***), 158.3 (C***_i_***), 134.4 (C***_c_***), 129.8 (C***_b_***), 129.5 (C***_k_***), 128.3 (C***_a_***), 126.3 (C***_d_***), 120.6 (Cl), 114.4 (C***_j_***), 65.7 (C***_h_***), 38.9 (C***_g_***).

**HR-ESI-MS:** m/z = 405.1808 [M+H]^+^ calc. 405.1809

**FTIR (neat)**: 3300.57 (w), 3063.37 (m), 2929.82 (m), 1636.79 (s), 1577.49 (m), 1537.47 (m), 1496.01 (m), 1464.67 (s),1457.92 (m), 1300.75 (m), 1236.63 (w), 1173.95 (m), 1115.14 (s), 1052.46 (s), 925.18 (m), 911.2 (s), 869.26 (m), 817.19 (s), 746.32 (s), 687.02 (w), 654.71 (w), 612.29 (m), 601.68 (s)

**M.p.**: 145.5 – 146.6 °C

Figure S96 – ^1^H NMR (DMSO-*d*_6_, 500 MHz) of **HM**

Figure S97 – JMOD ^13^C NMR (DMSO-*d*_6_, 126 MHz) of **HM**

Figure S98 – HMBC NMR (DMSO-*d*_6_) of **HM**

Figure S99 – HSQC NMR (DMSO-*d*_6_) of **HM**

Figure S100 – COSY NMR (DMSO-*d*_6_) of **HM**

Figure S101 - FTIR (neat) of **HM**

# 2 UV-Vis Characterisation

**Table S1 - An overview of all of the collected photoswitching data in different solvents. Solvents: *A* – MeCN:CHCl_3_ (1:1), *B* – CH_2_Cl_2_, *C* - PhMe**

|  | **λ_max_ E (nm)** | | | **ε at (λ_max_ E)**  **(L mol^-1^ cm^-1^)** | | | **λ_max_ PSS (nm)** | | | **ε at (λ_max_ PSS)**  **(L mol^-1^ cm^-1^)** | | | **%*Z* at the PSS at 25 °C (λ LED)** | | | **t_1/2_ at 25 °C (s)** | | | **Φ_(_*_E→Z_*_)_ (LED)** | | | **Φ_(Z_*_→E_*_)_ (LED)** | | |
| --- | --- | --- | --- | --- | --- | --- | --- | --- | --- | --- | --- | --- | --- | --- | --- | --- | --- | --- | --- | --- | --- | --- | --- | --- |
|  | *A* | *B* | *C* | *A* | *B* | *C* | *A* | *B* | *C* | *A* | *B* | *C* | *A* | *B* | *C* | *A* | *B* | *C* | *A* | *B* | *C* | *A* | *B* | *C* |
| **Me-Rot** | 661 | 647 | 642 | 4430 | 10632 | 7595 | 653 | 644 | 639 | 1830 | 5526 | 4117 | 10% 730 nm  19% 680 nm  40% 625 nm  22% 590 nm  23% 565 nm  16% 530 nm  12% 505 nm  19% 470 nm | 2% 730 nm  26% 680 nm  30% 625 nm  8% 590 nm  13% 565 nm  6% 530 nm  3% 505 nm  10% 470 nm | 3% 730 nm  28% 680 nm  45% 625 nm  19% 590 nm  15% 565 nm  9% 530 nm  5% 505 nm  13% 470 nm | 22.5 | 12.1 | 21.8 | 0.8%  (625 nm) | 12%  (625 nm) | 10%  (625 nm) | 38%  (625 nm) | 7%  (625 nm) | 14%  (625 nm) |
| **Boc-Rot** | 553 | 554 | 554 | 6350 | 4399 | 7488 | 542 | 554 | 554 | 3740 | 2784 | 2792 | 0% 730 nm  0% 680 nm  21% 625 nm  44% 590 nm  37% 565 nm  33% 530 nm  24% 505 nm  12% 470 nm | 0% 730 nm  0% 680 nm  0% 625 nm  30% 590 nm  36% 565 nm  32% 530 nm  23% 505 nm  8% 470 nm | 0% 730 nm  0% 680 nm  0% 625 nm  23% 590 nm  23% 565 nm  18% 530 nm  9% 505 nm  0% 470 nm | 3570 | 58560 | 35880 | 2%  (470 nm)  1%  (590 nm) | 2%  (470 nm)  0.3%  (590 nm) | 7%  (470 nm)  0.06%  (590 nm) | 4%  (470 nm)  3%  (590 nm) | 1%  (470 nm) 2%  (590 nm) | 6%  (470 nm) 0.5%  (590 nm) |
| **Me-Axle** | 650 | 641 | 641 | 8900 | 5273 | 4426 | 649 | 641 | 636 | 8970 | 4690 | 3877 | 0% 730 nm  0% 680 nm  20% 625 nm  6% 590 nm  0% 565 nm  0% 530 nm  0% 505 nm  0% 470 nm | 1% 730 nm  6% 680 nm  17% 625 nm  3% 590 nm  3% 565 nm  2% 530 nm  2% 505 nm  5% 470 nm | 2% 730 nm  17% 680 nm  23% 625 nm  11% 590 nm  10% 565 nm  5% 530 nm  4% 505 nm  8% 470 nm | 0.223 | 7.49 | 10.3 | 18%  (625 nm) | 9%  (625 nm) | 29%  (625 nm) | 3%  (625 nm) | 12%  (625 nm) | 37%  (625 nm) |
| **Boc-Axle** | 549 | 551 | 552 | 6080 | 5658 | 4192 | 516 | 545 | 517 | 3500 | 2165 | 1978 | 0% 730 nm  0% 680 nm  46% 625 nm  55% 590 nm  46% 565 nm  41% 530 nm  28% 505 nm  11% 470 nm | 0% 730 nm  0% 680 nm  5% 625 nm  20% 590 nm  44% 565 nm  40% 530 nm  29% 505 nm  12% 470 nm | 0% 730 nm  0% 680 nm  9% 625 nm  50% 590 nm  51% 565 nm  47% 530 nm  35% 505 nm  10% 470 nm | 85.8 | 24300 | 23220 | 7%  (470 nm)  5%  (590 nm) | 9%  (470 nm)  1%  (590 nm) | 3%  (470 nm)  2%  (590 nm) | 10%  (470 nm)  1%  (590 nm) | 1%  (470 nm) 2%  (590 nm) | 1%  (470 nm)  0.4%  (590 nm) |

## 2.2 Molar absorption coefficients

### 2.2.1 Procedure

The absorbance spectrum of a photoswitch solution was collected at concentrations of 100 μM, 90 μM, 80 μM, 70 μM, and 60 μM, Spectra were collected using Agilent Technology Scan Application. The λ_max_ of the π → π* transition was extracted and plotted against concentration to give the calibration curve.

Molar absorption coefficients were calculated according to Beer-Lambert Law.^[8]^ This can be expressed as:

$$\text{A}=\text{εlc}$$

*A – absorbance (a.u.), ε – molar absorption coefficient (mol^-1^dm^3^cm^-1^), l - optical path (cm) (fixed to 1 cm for all experiments), c – molar concentration of the sample (M).*

The molar absorption coefficient (ε) at λ_max_ is determined by the gradient of the calibration curve.

*E→Z* and ‘*Z→E*’ PSS used the same procedure under irradiation of the LED that gave the greatest %*Z* and %*E* for each specific photoswitch respectively.

### 2.2.2 Dark Extinction Molar Absorption Coefficient

Figure S102 – Calibration curve of **Me-Rot** (60 to 100 μM in MeCN:CHCl_3_ (1:1)). Absorbance measured at λ_max_ (662 nm) at 25 °C.

Figure S103 – Calibration curve of **Me-Rot** (20 to 100 μM in CH_2_Cl_2_). Absorbance measured at λ_max_ (647 nm) at 25 °C.

Figure S104 – Calibration curve of **Me-Rot** (20 to 100 μM in PhMe). Absorbance measured at λ_max_ (642 nm) at 25 °C.

Figure S105 - Calibration curve of **Boc-Rot** (60 to 100 μM in MeCN:CHCl_3_ (1:1)). Absorbance measured at λ_max_ (553 nm) at 25 °C.

Figure S106 – Calibration curve of **Boc-Rot** (20 to 100 μM in CH_2_Cl_2_). Absorbance measured at λ_max_ (554 nm) at 25 °C.

Figure S107 – Calibration curve of **Boc-Rot** (20 to 100 μM in PhMe). Absorbance measured at λ_max_ (554 nm) at 25 °C.

Figure S108 - Calibration curve of **Me-Axle** (60 to 100 μM in MeCN:CHCl_3_ (1:1)). Absorbance measured at λ_max_ (650 nm) at 25 °C.

Figure S109 – Calibration curve of **Me-Axle** (20 to 100 μM in CH_2_Cl_2_). Absorbance measured at λ_max_ (641 nm) at 25 °C.

Figure S110 – Calibration curve of **Me-Axle** (20 to 100 μM in PhMe). Absorbance measured at λ_max_ (641 nm) at 25 °C.

Figure S111 – Calibration curve of **Boc-Axle** (60 to 100 μM in MeCN:CHCl_3_ (1:1)). Absorbance measured at λ_max_ (549 nm) at 25 °C.

Figure S112 – Calibration curve of **Boc-Axle** (20 to 100 μM in CH_2_Cl_2_). Absorbance measured at λ_max_ (551 nm) at 25 °C.

Figure S113 – Calibration curve of **Boc-Axle** (20 to 100 μM in PhMe). Absorbance measured at λ_max_ (552 nm) at 25 °C.

### 2.2.3 Forward PSS Molar Absorption Coefficient

Figure S114 – Calibration curve of **Me-Rot** (60 to 100 μM in MeCN:CHCl_3_ (1:1)) under irradiation of 625 nm light. Absorbance measured at λ_max_ (653 nm) at 25 °C.

Figure S115 – Calibration curve of **Me-Rot** (20 to 100 μM in CH_2_Cl_2_) under irradiation of 625 nm light. Absorbance measured at λ_max_ (644 nm) at 25 °C.

Figure S116 – Calibration curve of **Me-Rot** (20 to 100 μM in PhMe) under irradiation of 625 nm light. Absorbance measured at λ_max_ (639 nm) at 25 °C.

Figure S117 – Calibration curve of **Boc-Rot** (60 to 100 μM in MeCN:CHCl_3_ (1:1)) under irradiation of 590 nm light. Absorbance measured at λ_max_ (545 nm) at 25 °C.

Figure S118 – Calibration curve of **Boc-Rot** (20 to 100 μM in CH_2_Cl_2_) under irradiation of 590 nm light. Absorbance measured at λ_max_ (554 nm) at 25 °C.

Figure S119 – Calibration curve of **Boc-Rot** (20 to 100 μM in PhMe) under irradiation of 590 nm light. Absorbance measured at λ_max_ (554 nm) at 25 °C.

Figure S120 – Calibration curve of **Me-Axle** (60 to 100 μM in MeCN:CHCl_3_ (1:1)) under irradiation of 625 nm light. Absorbance measured at λ_max_ (649 nm) at 25 °C.

Figure S121 – Calibration curve of **Me-Axle** (20 to 100 μM in CH_2_Cl_2_) under irradiation of 625 nm light. Absorbance measured at λ_max_ (641 nm) at 25 °C.

Figure S122 – Calibration curve of **Me-Axle** (20 to 100 μM in PhMe) under irradiation of 625 nm light. Absorbance measured at λ_max_ (636 nm) at 25 °C.

Figure S123 – Calibration curve of **Boc-Axle** (60 to 100 μM in MeCN:CHCl_3_ (1:1)) under irradiation of 590 nm light. Absorbance measured at λ_max_ (516 nm) at 25 °C.

Figure S124 – Calibration curve of **Boc-Axle** (20 to 100 μM in CH_2_Cl_2_) under irradiation of 590 nm light. Absorbance measured at λ_max_ (545 nm) at 25 °C.

Figure S125 – Calibration curve of **Boc-Axle** (20 to 100 μM in PhMe) under irradiation of 590 nm light. Absorbance measured at λ_max_ (517 nm) at 25 °C.

### 2.2.4 Back PSS Molar Absorption Coefficient

Figure S126 - Calibration curve of **Boc-Rot** (60 to 100 μM in MeCN:CHCl_3_ (1:1)) under irradiation of 470 nm light. Absorbance measured at λ_max_ (518 nm) at 25 °C.

Figure S127 - Calibration curve of **Boc-Axle** (60 to 100 μM in MeCN:CHCl_3_ (1:1)) under irradiation of 470 nm light. Absorbance measured at λ_max_ (518 nm) at 25 °C.

## 2.3 Photostationary states

### 2.3.1 Procedure

Each photostationary state was determined by irradiating a photoswitch solution with an LED and monitoring the change in the absorbance spectrum. The spectrum was observed at regular time intervals until it ceased changing. The final spectrum was then collected, giving the PSS spectrum at that irradiation wavelength. This was performed for a range of LEDs (starting with the longest wavelength, ending with the shortest) within which changes in absorbance spectra could be observed in at least one of an axle-rotaxane pairing. The experiments were monitored using Agilent Technologies Scanning application.

The calculations of PSS ratio (%PSS) were performed according to procedure outlined by Huang *et al*.^[9]^ The proportion of *E*-isomers (%*E*) and the proportion of *Z*-isomers (%*Z*) at a PSS can be estimated by the relationship between the dark spectra (Pure *E*) and the PSS spectra.

Abs(PSS)/Abs(*E*) was plotted against wavelength and the region where the plot is horizontal gives %*E*. An example is displayed below for **Me-Rot** under irradiation of 730 nm light.

The area where the curve remains horizontal denotes the region where only the *E*-isomer absorbs but not the *Z*-isomer. Consequently, this ratio represents the %*E*. The %*Z* can be calculated by the following relationship:

$$\%\text{Z}=100-\%\text{E}$$

With this number, the pure *Z*-spectrum can be deduced using:

$$\text{Abs}\left( \text{Z} \right)=\frac{\text{Abs}\left( \text{PSS} \right)-\%\text{E}\times\text{Abs}\left( \text{E} \right)}{\%\text{Z}}$$

The calculated pure *Z*-spectrum was then smoothed using a Savitzky–Golay filter with 5 points of window and a polynomial order of 2.^[10]^

### 2.3.2  Spectra

Figure S128 – UV-Vis spectra of the PSSs and calculated 100% *Z*-isomer of **Me-Rot** (220 μM, MeCN:CHCl_3_ (1:1)) at 25 °C following irradiation with LEDs.

Figure S129 – UV-Vis spectra of the PSSs and calculated 100% *Z*-isomer of **Me-Rot** (80 μM, CH_2_Cl_2_) at 25 °C following irradiation with LEDs.

Figure S130 – UV-Vis spectra of the PSSs and calculated 100% *Z*-isomer of **Me-Rot** (80 μM, PhMe) at 25 °C following irradiation with LEDs.

Figure S131 – UV-Vis spectra of the PSSs and calculated 100% *Z*-isomer of **Boc-Rot** (85 μM, MeCN:CHCl_3_ (1:1)) at 25 °C following irradiation with LEDs.

Figure S132 – UV-Vis spectra of the PSSs and calculated 100% *Z*-isomer of **Boc-Rot** (80 μM, CH_2_Cl_2_) at 25 °C following irradiation with LEDs.

Figure S133 – UV-Vis spectra of the PSSs and calculated 100% *Z*-isomer of **Boc-Rot** (80 μM, PhMe) at 25 °C following irradiation with LEDs.

Figure S134 – UV-Vis spectra of the PSSs and calculated 100% *Z*-isomer of **Me-Axle** (100 μM, MeCN:CHCl_3_ (1:1)) at 25 °C following irradiation with LEDs.

Figure S135 – UV-Vis spectra of the PSSs and calculated 100% *Z*-isomer of **Me-Axle** (80 μM, CH_2_Cl_2_) at 25 °C following irradiation with LEDs.

Figure S136 – UV-Vis spectra of the PSSs and calculated 100% *Z*-isomer of **Me-Axle** (80 μM, PhMe) at 25 °C following irradiation with LEDs.

Figure S137 – UV-Vis spectra of the PSSs and calculated 100% Z-isomer of **Boc-Axle** (150 μM, MeCN:CHCl_3_ (1:1)) at 25 °C following irradiation with LEDs.

Figure S138 – UV-Vis spectra of the PSSs and calculated 100% Z-isomer of **Boc-Axle** (80 μM, CH_2_Cl_2_) at 25 °C following irradiation with LEDs.

Figure S139 – UV-Vis spectra of the PSSs and calculated 100% Z-isomer of **Boc-Axle** (80 μM, PhMe) at 25 °C following irradiation with LEDs.

## 2.4 Forward Switching (*E* → *Z*)

### 2.4.1 Procedure

A solution of a photoswitch was irradiated using the LED that gave the greatest %*Z* until at PSS. The changing absorbance spectra was measured over specified time periods using Agilent Technology Scanning Kinetics application.

### 2.4.2 Spectra

Figure S140 - *E* to *Z* switching of **Me-Rot** (100 μM in MeCN:CHCl_3_ (1:1)) under 625 nm irradiation at 25 °C.

Figure S141 - *E* to *Z* switching of **Boc-Rot** (100 μM in MeCN:CHCl_3_ (1:1)) under 590 nm irradiation at 25 °C.

Figure S142 - *E* to *Z* switching of **Me-Axle** (100 μM in MeCN:CHCl_3_ (1:1)) under 625 nm irradiation at 25 °C.

Figure S143 - *E* to *Z* switching of **Boc-Axle** (140 μM in MeCN:CHCl_3_ (1:1)) under 590 nm irradiation at 25 °C.

## 2.5 Back Switching (*Z* → *E*)

### 2.5.1 Procedure

A solution of photoswitch was either irradiated using the LED that gave the greatest %*E* until at PSS or allowed to thermally revert to the dark state – whichever was the quicker process. The changing absorbance spectra was measured over specified time periods using Agilent Technology Scanning Kinetics application.

### 2.5.2 Spectra

Figure S144 - *Z* to *E* switching of **Me-Rot** (100 μM in MeCN:CHCl_3_ (1:1)) thermally at 25 °C.

Figure S145 - *Z* to *E* switching of **Boc-Rot** (100 μM in MeCN:CHCl_3_ (1:1)) at 25 °C under 470 nm irradiation.

Figure S146 - *Z* to *E* switching of **Me-Axle** (100 μM in MeCN:CHCl_3_ (1:1)) thermally at 25 °C.

Figure S147 - *Z* to *E* switching of **Boc-Axle** (140 μM in MeCN:CHCl_3_ (1:1)) at 25 °C under 470 nm irradiation.

## 2.6 Thermal reversion

### 2.6.1 Procedure

Each compound was irradiated by the LED that gave the greatest %*Z* until it reached the PSS. Following this, the LED was turned off and the absorbance at the λ_max_ was then measured at regular time points using Agilent Technologies Kinetics application.

Assuming a first-order reaction, the rate constants (k) for the thermodynamic switching process from the *Z*- to *E*-isomer were determined by fitting the absorbance changes over time to the negative exponential curve for first-order reactions:

$$\text{Abs}_{\text{t}}=-\text{Ab}\text{s}_{\text{0}}\times e^{\left( -\left( \text{k}\times\text{t} \right) \right)}+\text{c}$$

*Abs_t_ – Absorbance at Time = t (a.u.), Abs_0_ – Max Absorbance (a.u), k – Rate Constant (mins^-1^), t – Time (mins), c – Minimum Absorbance (a.u)*

Using OriginPro 2020 non-linear curve fit function, k was able to be determined. The thermal half-lives (t_1/2_) were then calculated based on the relationship:

$$\text{t}_{\frac{\text{1}}{\text{2}}}=\frac{\ln\left( 2 \right)}{\text{k}}$$

### 2.6.2 Spectra

Figure S148 – Thermal Reversion of **Me-Rot** (20 μM, MeCN:CHCl_3_ (1:1)) at 25 °C

Figure S149 – Thermal Reversion of **Me-Rot** (80 μM, CH_2_Cl_2_) at 25 °C

Figure S150 – Thermal Reversion of **Me-Rot** (80 μM, PhMe) at 25 °C

Figure S151 – Thermal Reversion of **Boc-Rot** (150 μM, MeCN:CHCl_3_ (1:1)) at 25 °C

Figure S152 – Thermal Reversion of **Boc-Rot** (80 μM, CH_2_Cl_2_) at 25 °C

Figure S153 – Thermal Reversion of **Boc-Rot** (80 μM, PhMe) at 25 °C

Figure S154 – Thermal Reversion of **Me-Axle** (100 μM, MeCN:CHCl_3_ (1:1)) at 25 °C

Figure S155 – Thermal Reversion of **Me-Axle** (25 μM,CH_2_Cl_2_) at 25 °C

Figure S156 – Thermal Reversion of **Me-Axle** (25 μM,PhMe) at 25 °C

Figure S157 – Thermal Reversion of **Boc-Axle** (150 μM, MeCN:CHCl_3_ (1:1)) at 25 °C

Figure S158 – Thermal Reversion of **Boc-Axle** (100 μM, CH_2_Cl_2_) at 25 °C

Figure S159 – Thermal Reversion of **Boc-Axle** (20 μM, PhMe) at 25 °C

## 2.7 Quantum yield determination

### 2.7.1 Actinometry

To obtain the quantum yields of the set up outlined in **1.1**, the incident photon flux upon the samples for each LED that provided a greatest %*Z* or greatest %*E* was required (470 nm, 590 nm, and 625 nm). This was determined using a chemical actinometer. Chemical actinometry was performed according to the procedures from Cornet *et al*.^[11]^ and Radjagobalou *et al*.^[12]^

#### 2.7.1.1 Preparation of Actinometry Solutions

A 0.015 M solution of Reinecke’s salt was prepared in KOH solution (aq., 0.1 M) in the dark and stirred vigorously for 1 hour. The solution was then acidified by the slow addition of sulfuric acid (aq., conc.) until at pH 4. Separately, a 0.1 M solution of ferrous nitrate nonahydrate was prepared in nitric acid solution (aq., 0.5 M); these solutions were made fresh for each reading.

#### 2.7.1.2 Collection of Actinometry Data

In a dark room, 3 mL of the Reinecke’s salt solution was added to a quartz cuvette and irradiated with the chosen LED in the set position outlined in **S1.1** for defined time intervals, with the current controlled by a Prizmatix Pulser Plus. At each time point, 0.5 mL of irradiated solution was mixed with 1.5 mL of ferrous nitrate solution and diluted to 12 mL with distilled HPLC grade H_2_O and the absorbance spectra collected between 350 – 700 nm to extract the absorbance at 450 nm (Abs_450 nm_) which was then plotted versus the time of collection. This, alongside the quantum yield (0.29) and extinction coefficient of Reinecke’s salt solution (3400 mol^-1^ dm^3^ cm^-1^), were analysed by MatLab software provided by Stranius and Börjesson to provide the photon flux in photons per second (photon s^-1^) in the set up at the desired current.^[13]^

Figure S160 - Determination of the photon flux absorbance spectra and Linear best fit of Absorption vs Time (seconds) using Reinecke’s salt, A) 470 nm (1 A), B) 590 nm (1 A), C) 625 nm (1 A),

Table S2 – Summary of the photon flux of the utilised LEDs through the Reinecke’s salt method.

| **LED λ (nm)** | **Current (A)** | **Photon flux (photons s^-1^)** |
| --- | --- | --- |
| **470** | 1 | 3.453726 x 10^15^ |
| **590** | 1 | 2.587521 x 10^15^ |
| **625** | 1 | 2.322052 x 10^15^ |

### 2.7.2 Determining Quantum yields

Using the photon fluxes of the LEDs that provided the greatest %*Z* determined, the method outlined by Stranius and Börjesson was employed.^[13]^ Quantum yields were calculated using the provided calculator from their publication.

$$\frac{d\left[ A \right]}{dt}=-\frac{\phi_{A\to B}\cdot I\cdot\beta_{A}\left( t \right)}{N_{A}\cdot V}+\frac{\phi_{B\to A}\cdot I\cdot\beta_{B}\left( t \right)}{N_{A}\cdot V}+k_{t, B\to A}$$

Where, $\frac{d\left[ A \right]}{dt}$ is the rate of change of absorbance, $\phi_{A\to B}$ is the quantum yield of the forward reaction, $\phi_{B\to A}$ is the quantum yield of the reverse reaction, $I$ is the photonflux, $\beta_{A}$ is the fraction of photons absorbed by the stable state, $\beta_{B}$ is the fraction of photons absorbed by the metastable state., $N_{A}$ is Avogadro’s number, $V$ is the volume of the sample (dm^3^), and $k_{t, B\to A}$ is the thermal reversion rate constant (S^-1^).

Figure S161 – The switching rates for **Me Rot**, monitored at 625 nm under irradiation of 625 nm light. (Left to right: 100 μM, MeCN:CHCl_3_ (1:1); 100 μM, CH_2_Cl_2_ ; 100 μM, PhMe)

Figure S162 – The switching rates for **Boc Rot**, monitored at 590 nm under irradiation of 590 nm light. (Left to right: 100 μM, MeCN:CHCl_3_ (1:1); 100 μM, CH_2_Cl_2_ ; 100 μM, PhMe)

Figure S163 – The switching rates for **Boc Rot**, monitored at 470 nm under irradiation of 470 nm light. (Left to right: 100 μM, MeCN:CHCl_3_ (1:1); 100 μM, CH_2_Cl_2_ ; 100 μM, PhMe)

Figure S164 – The switching rates for **Me Axle**, monitored at 625 nm under irradiation of 625nm light. (Left to right: 100 μM, MeCN:CHCl_3_ (1:1); 50 μM, CH_2_Cl_2_ ; 50 μM, PhMe)

Figure S165 – The switching rates for **Boc Axle**, monitored at 590 nm under irradiation of 590 nm light. (Left to right: 100 μM, MeCN:CHCl_3_ (1:1); 100 μM, CH_2_Cl_2_ ; 100 μM, PhMe)

Figure S166 – The switching rates for **Boc Axle**, monitored at 470 nm under irradiation of 470 nm light. (Left to right: 100 μM, MeCN:CHCl_3_ (1:1); 100 μM, CH_2_Cl_2_ ; 100 μM, PhMe)

Table S3 – Summary of the quantum yields of the *E*→*Z* and *Z*→*E* quantum yields for each of the compounds.

|  | **Φ_(_*_E→Z_*_)_ (LED)** | | | **Φ_(Z_*_→E_*_)_ (LED)** | | |
| --- | --- | --- | --- | --- | --- | --- |
| **Solvent**  **Compound** | MeCN:CHCl_3_ (1:1) | CH_2_Cl_2_ | PhMe | MeCN:CHCl_3_ (1:1) | CH_2_Cl_2_ | PhMe |
| **Me-Rot** | 0.8%  (625 nm) | 12%  (625 nm) | 10%  (625 nm) | 38%  (625 nm) | 7%  (625 nm) | 14%  (625 nm) |
| **Boc-Rot** | 2%  (470 nm)  1%  (590 nm) | 2%  (470 nm)  0.3%  (590 nm) | 7%  (470 nm)  0.06%  (590 nm) | 4%  (470 nm)  3%  (590 nm) | 1%  (470 nm) 2%  (590 nm) | 6%  (470 nm) 0.5%  (590 nm) |
| **Me-Axle** | 18%  (625 nm) | 9%  (625 nm) | 29%  (625 nm) | 3%  (625 nm) | 12%  (625 nm) | 37%  (625 nm) |
| **Boc-Axle** | 7%  (470 nm)  5%  (590 nm) | 9%  (470 nm)  1%  (590 nm) | 3%  (470 nm)  2%  (590 nm) | 10%  (470 nm)  1%  (590 nm) | 1%  (470 nm) 2%  (590 nm) | 1%  (470 nm)  0.4%  (590 nm) |

## 2.8 Fatigue studies

### 2.8.1 Procedure

A solution of photoswitch was irradiated using the LED that gave the greatest %*Z* until at PSS. They were then either irradiated with the LED that gave the greatest %*E* or thermally reverted, whichever was the quicker process. This was repeated for 10 cycles.

The absorbance at λ_max_ was recorded at regular time intervals using Agilent Technologies Kinetics application. The maximum and minimum absorbance of each cycle were then extracted and plotted versus number of cycles to give the fatigue plot.

### 2.8.2 Data

Figure S167 – Fatigue study of **Me-Rot** (125 μM, MeCN:CHCl_3_ (1:1)). Irradiated with 625 nm then thermally reverted at 25 °C, monitoring the absorbance at 662 nm.

Figure S168 - Fatigue study of **Boc-Rot** (75 μM, MeCN:CHCl_3_ (1:1)). Irradiated with 590 nm then 470 nm at 25 °C, monitoring the absorbance at 553 nm.

Figure S169 - Fatigue study of **Me-Rot** (100 μM, MeCN:CHCl_3_ (1:1)). Irradiated with 625 nm the thermally reverted at 25 °C, monitoring the absorbance at 650 nm.

Figure S170 – Fatigue study of **Boc-Rot** (150 μM, MeCN:CHCl_3_ (1:1)). Irradiated with 590 nm and 470 nm at 25 °C, monitoring the absorbance at 549 nm.

# 3 Non-Interlocked Control Experiments

## 3.1 Absorbance Spectra

### 3.1.1 Procedure

The absorbance spectrum of an axle solution was collected using Agilent Technology Scan Application using solutions of the Axle and Rotaxane compounds in the presence of 10 equivalents of **HM** in MeCN:CHCl_3_ (1:1).

### 3.1.2 Spectra

Figure S171 – The absorbance spectra of **Me-Axle** (80 μM, MeCN:CHCl_3_ (1:1)) before (black) and after (green) the addition of 10 equivalents of **HM**.

Figure S172 – The absorbance spectra of **Boc-Axle** (80 μM, MeCN:CHCl_3_ (1:1)) before (black) and after (green) the addition of 10 equivalents of **HM**.

## 3.2 Photostationary states

### 3.2.1 Procedure

The procedure follows that outlined in **3.2.1**, irradiating only the wavelength that gave the greatest %*Z* upon a solution of an axle in the presence of 10 equivalents of **HM**.

### 3.2.2 Spectra

Figure S173 – UV-Vis spectra of **Me-Axle** (80 μM, MeCN:CHCl_3_ (1:1)) + **HM** (10 eq.) at 25 °C before and after irradiation with 625 nm light.

Figure S174– UV-Vis spectra of **Boc-Axle** (80 μM, MeCN:CHCl_3_ (1:1)) + **HM** (10 eq.) at 25 °C before and after irradiation with 625 nm light.

## 3.3 Thermal Reversion

### 3.3.1 Procedure

The procedure follows **3.5.1**, with the axle in the presence of **HM** (10 eq.).

### 3.3.2 Spectra

Figure S175 – Thermal reversion of **Me-Axle** (80 μM, MeCN:CHCl_3_ (1:1)) + **HM** (10 eq.) at 25 °C

Figure S176– Thermal reversion of **Boc-Axle** (80 μM, MeCN:CHCl_3_ (1:1)) + **HM** (10 eq.) at 25 °C

# 4 Computational studies

## 4.1 Conformational analysis

Conformational analysis was performed using CREST (Conform-Rotamer Ensemble Sampling Tool) v3.0.2 ^[14]^[1], employing the GFN-FF force field [2].^[15]^ The resulting conformer ensembles were then re-optimized at the GFN2-xTB level of theory [3]^[16, 17]^ with the ALPB (acetonitrile) implicit solvation model [4],^[18]^ and subsequently filtered using the CREGEN (Conformer–Rotamer Ensemble GENeration) tool included in the CREST package. Electronic energies (E_elec_) of the structures were corrected using the PBEh-3c composite density functional method with SMD (acetonitrile) [5].^[19]^ Thermal corrections (G_thermo_) were obtained at the same GFN2-xTB level with xTB v6.5.1. Final Gibbs free energies for each conformer were obtained from:

*G_Gibb_= E_elec_ + G_thermo_*

with the lowest-energy conformer within each set taken as the reference.

The cartesian coordinates are stored in a figshare database under the following DOI: [doi.org/10.6084/m9.figshare.31045387](https://doi.org/10.6084/m9.figshare.31045387)

Figure S177 - (a) Illustration of the N-H···O hydrogen-bond distances between the macrocycle and the axle in **Me-Rot**. List of the distances and the relative Gibbs free energies of conformers for (b) **Me-Rot***_E_*_-A_ and (c) **Me-Rot***_Z_*_-A_.

Figure S178 - (a) Illustration of the N-H···O hydrogen-bond distances between the macrocycle and the axle in **Boc-Rot**. List of the distances and the relative Gibbs free energies of conformers for (b) **Boc-Rot***_E_*_-A_ and (c) **Boc-Rot***_Z_*_-A_.

## 4.2 Excited state calculations

Initially, all point structures were generated through ground-state potential energy surface (PES) scans at the GFN2-xTB level using the xTB software.^[16,17]^ Subsequently, each point structure obtained from the ground-state scans was reoptimized in the first excited state using the GFN0-xTB* level of theory to obtain improved excited-state geometries employing the CREST software.^14^ During the proton-transfer scans, constraints were imposed on the N–C–C–N dihedral angle to prevent rotational motion during proton transfer. Additionally, constraints on the N···O distance were applied to avoid artificial N–O bond formation, particularly in the *cis*-conformation region. The same geometrical constraints were retained during the excited-state optimizations to generate consistent excited-state PESs.

Single-point energy calculations were then carried out for each optimized point structure using spin-flip density functional theory (SF-DFT) to obtain more accurate relative energies employing the ORCA quantum chemistry package (Version 6.1.1).^[20]^ These SF-DFT calculations were performed using an ONIOM-like multilayer approach, in which the active region consisting of 64 out of 151 atoms was treated at the BHLYP hybrid functional in conjunction with Def2-TZVP basis set, while the remaining atoms were described using the xTB level of theory. This method was selected because previous benchmark studies have demonstrated reliable SF-DFT performance for functionals containing a larger fraction of Hartree–Fock exchange.^[21,22]^ During the SF-DFT calculations, Grimme’s D4 dispersion correction and geometrical counterpoise (gCP) correction were included.^[21,22]^ To reduce computational cost and complexity, the bulky substituents at the termini of the photoswitch moiety were omitted from the calculations. The omitted substituents are not anticipated to substantially affect the mechanistic aspects of proton transfer and hydrogen bonding interactions associated with the photoisomerization process under investigation.

In the photoswitch-encapsulated rotaxane architecture, the distances between the rotaxane N–H groups and the O atom of the photoswitch fall within the typical hydrogen-bonding range. Therefore, intermolecular interactions, such as strengthened hydrogen bonding and possible proton transfer, may play an important role during the photoisomerization process. To investigate this possibility, computational studies were performed to explore the photoisomerization pathway together with the associated proton-transfer process. Potential energy surfaces (PESs) corresponding to both the *E*-to-*Z* photoisomerization pathway and proton transfer from the rotaxane N–H group to the O atom of the photoswitch were generated. The resulting ground- and excited-state PESs are shown in Figure S179. A few critical geometries together with key structural parameters and relative energies on the excited PES are highlighted in the same figure.

The calculations reveal that the N–H groups form stable hydrogen bonding interactions with the O atom of the photoswitch throughout the isomerization pathway. On the excited-state PES, the critical-point structure Iso-1, characterized by an N–C=C–N distances of 1.94 and 1.59 Å corresponds to minimum-energy geometry. However, proton transfer from the N–H group to the O atom, leading to a shorter O···H distance of 1.16 Å (Iso-2), requires overcoming an activation barrier of 17.7 kcal mol⁻^1^. Similarly, proton transfer along the ground-state isomerization pathway is associated with a substantial activation barrier (43.7 kcal mol⁻^1^ for *Z* and 37.8 kcal mol⁻^1^ for *E*). These results indicate that complete proton transfer is energetically unfavorable in both electronic states.

Despite the absence of facile proton transfer, strong hydrogen bonding interactions persist along the entire photoisomerization pathway, as evidenced by the geometrical parameters shown in Figure S179a. An important question arising from these observations is whether both N–H groups participate cooperatively in hydrogen bonding or whether one interaction dominates the process. To address this, along with the PES calculations, we also analysed the conical intersection (CI) geometry optimized at the same level of theory as the stationary points on the excited state PES. At the CI geometry, both O–H distances are 1.59 Å (Figure S180), while the N–H distances remain close to 1.04 Å. The optimized CI geometry is found to be closer to the Iso-3 structure (Figure S179) located on the excited-state PES generated for analysing the H-transfer-associated photoisomerization process. The geometrical features of the CI suggest a significant strengthening of both hydrogen-bonding interactions. This further indicates that both protons remain associated with their respective nitrogen atoms and that complete proton transfer does not occur at the CI, as it is energetically demanding. Collectively, these findings provide clues that, rather than involving complete hydrogen migration, the enhanced hydrogen-bonding interactions involving both hydrogen atoms may play a crucial role during the photoisomerization process within the confined rotaxane environment.

| **a)** | | |
| --- | --- | --- |
| **b)** | | |
|  |  |  |
| **Iso-1 (0.0)** | **Iso-2 (17.7)** | **Iso-3 (2.5)** |

Figure S179 - (a) Illustration Potential energy surface for the isomerization of the photoswitch within the rotaxane (a), together with the critical point structures on the excited-state PES (b) highlighting the key hydrogen-bonding interactions, N–H and O···H bond distances (Å), and relative energies (kcal mol^-1^).

**CI-geometry**

Figure S180 - Optimized geometry of the conical intersection (CI) illustrating the enhanced hydrogen-bonding interactions arising from the reduced O···H distances.

# 5 References

[1]	Mounted LEDs, https://www.thorlabs.com/newgrouppage9.cfm?objectgroup_id=2692, (accessed October 2025).

[2]	L. Holoidovsky, M. M. Meijler, *ACS Infect. Dis.* **2020**, *6*, 572–576.

[3]	M. Pandrala, A. Resendez, S. V. Malhotra, *J. Catal.* **2019**, *378*, 283–288.

[4]	Y. Yamamoto, Y. Inoue, U. Takaki, H. Suzuki, *Bull. Chem. Soc. Jap.* **2011**, *84*, 82–89.

[5]	O. K. Rasheed, P. D. Bailey, A. Lawrence, P. Quayle, J. Raftery, *Eur. J. Org. Chem.* **2015**, *2015*, 6988–6993.

[6]	T. Darbre, C. Dubs, E. Rusanov, H. Stoeckli-Evans, *Eur. J. Inorg. Chem.* **2002**, *2002*, 3284–3291.

[7]	A. Brown, T. Lang, K. M. Mullen, P. D. Beer, *Org. Biomol. Chem.* **2017**, *15*, 4587–4594.

[8]	*The IUPAC Compendium of Chemical Terminology: The Gold Book*, 5 ed., International Union of Pure and Applied Chemistry (IUPAC), Research Triangle Park, North Carolina, **2025**.

[9]	C.-Y. Huang, A. Bonasera, L. Hristov, Y. Garmshausen, B. M. Schmidt, D. Jacquemin, S. Hecht, *J. Am. Chem. Soc.* **2017**, *139*, 15205–15211.

[10]	Ľ. Vetráková, V. Ladányi, J. Al Anshori, P. Dvořák, J. Wirz, D. Heger, *Photochem Photobiol. Sci.* **2017**, *16*, 1749–1756.

[11]	J.-F. Cornet, A. Marty, J.-B. Gros, *Biotechnol. Prog.* **1997**, *13*, 408–415.

[12]	R. Radjagobalou, J.-F. Blanco, V. Dias da Silva Freitas, C. Supplis, F. Gros, O. Dechy-Cabaret, K. Loubière, *J. Photochem. Photobiol., A* **2019**, *382*, 111934.

[13]	K. Stranius, K. Börjesson, *Sci. Rep.* **2017**, *7*, 41145.

[14]	P. Pracht, S. Grimme, C. Bannwarth, F. Bohle, S. Ehlert, G. Feldmann, J. Gorges, M. Müller, T. Neudecker, C. Plett, S. Spicher, P. Steinbach, P. A. Wesołowski, F. Zeller, *J. Chem. Phys.* **2024**, *160*.

[15]	S. Spicher, S. Grimme, *Angew. Chem. Int. Ed.* **2020**, *59*, 15665–15673.

[16]	C. Bannwarth, E. Caldeweyher, S. Ehlert, A. Hansen, P. Pracht, J. Seibert, S. Spicher, S. Grimme, *WIREs Comp. Mol. Sci.* **2021**, *11*, e1493.

[17]	C. Bannwarth, S. Ehlert, S. Grimme, *J. Chem. Theory Comput.* **2019**, *15*, 1652–1671.

[18]	S. Ehlert, M. Stahn, S. Spicher, S. Grimme, *J. Chem. Theory Comput.* **2021**, *17*, 4250–4261.

[19]	S. Grimme, J. G. Brandenburg, C. Bannwarth, A. Hansen, *J. Chem. Phys.* **2015**, *143*.

[20]	F. Neese, *WIREs Comp. Mol. Sci.,* **2025**, *15*, e70019.

[21]	Y. Shao, M. Head-Gordon, A. I. Krylov, *J. Chem. Phys.* **2003**, *118*, 4807–4818.

[22]	N. Minezawa, M. S. Gordon, *J. Phys. Chem. A* **2009**, *113*, 12749–12753.

[23]	S. Grimme, *WIREs Comp. Mol. Sci.* **2011**, *1*, 211–228.

[24]	H. Kruse, S. Grimme, *J. Chem. Phys.* **2012**, *136*, 154101.
